# Aged ZnO Nanoparticles Promote Breast Cancer Lung Metastasis by Remodeling the Pulmonary Microenvironment Through the CXCL10/Wnt/β‐Catenin Axis

**DOI:** 10.1002/advs.78249

**Published:** 2026-10-11

**Authors:** Yong Zhu, Siyu Wang, Tong Lin, Jingyi Zhang, Jingya Yang, Dixiang Kang, Wenting Zhao, Na Yu, Meimei Wang

**Affiliations:** ^1^ Department of Pathophysiology School of Basic Medical Science Anhui Medical University Hefei Anhui P. R. China

**Keywords:** breast cancer metastasis, CXCL10, environmental transformation of nanoparticles, tumor microenvironment, Wnt/β‐catenin signaling pathway, zinc oxide nanoparticles

## Abstract

Our previous work demonstrated that zinc oxide nanoparticles (ZnO NPs) undergo physicochemical transformations during environmental aging, forming a composite mixture of zinc hydroxide carbonate, nanosized ZnO, and Zn^2+^ (termed aged ZnO NPs). Compared to their pristine counterparts, aged ZnO NPs exhibit significantly enhanced mutagenicity and malignant transformation capacity in mouse embryonic fibroblasts. However, their potential role in cancer initiation and progression in vivo remains unresolved. Here, we provide the first evidence that subchronic pulmonary exposure to aged ZnO NPs markedly accelerates lung metastasis and colony formation in a mouse model of breast cancer. Single‐cell transcriptomic profiling (scRNA‐seq) revealed that aged NPs remodel the metastatic microenvironment by recruiting M2 macrophages and myeloid‐derived suppressor cells, and by activating cancer‐associated fibroblasts. These findings were corroborated by analysis of public scRNA‐seq datasets. Mechanistically, CXCL10 derived from macrophages and fibroblasts was identified as a key mediator of metastasis via activation of the Wnt/β‐catenin pathway. Notably, pharmacological inhibition of the CXCL10/CXCR3 axis effectively attenuated NP‐induced metastasis. Collectively, this study uncovers the cellular and molecular mechanisms by which aged ZnO NPs potentiate breast cancer metastasis, highlighting the need to incorporate environmental aging processes into the risk assessment of nanomaterials.

## Graphical abstract

In summary, pulmonary exposure to ZnO NPs, particularly aged NPs, promotes breast cancer metastasis by remodelling the lung tumour microenvironment. Aged ZnO NPs enrich M2‐macrophages, MDSCs and activated CAFs to form an immunosuppressive metastatic niche. CXCL10 from these cells drives metastasis via Wnt/β‐catenin activation. This work reveals underlying mechanisms and stresses environmental‐aging factors in nanomaterial risk evaluation.

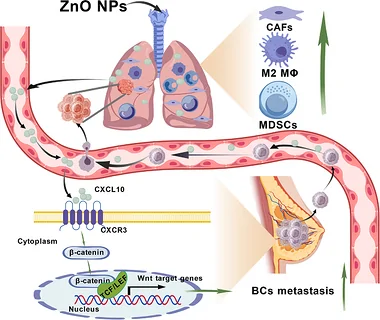

## 1 Introduction

With the extensive utilization of zinc oxide nanoparticles (ZnO NPs) in coatings, food preservation, personal care products, and biomedical applications, their potential health risks are no longer confined to occupational settings but have become a concern for the general population [1, 2, 3, 4]. In addition to the direct release of engineered ZnO NPs, atmospheric zinc emissions from incinerators, vehicular exhaust, and industrial processes predominantly exist in the form of ZnO NPs [5]. These aerosolized NPs from diverse sources represent a notable component of airborne particulate matter [6, 7]. Importantly, atmospheric ZnO NPs have been shown to be unstable‐ undergoing significant physicochemical transformations under specific pH conditions or through interactions with environmental pollutants [8]. Following inhalation, ZnO NPs may further transform upon contact with various biological fluids [7, 9, 10, 11]. Such transformations, occurring both extracellularly and within the human body, can substantially modify the biological behavior and toxicity of ZnO NPs, potentially introducing unforeseen health hazards. Therefore, the public is more likely to be exposed to transformed NPs rather than their pristine counterparts. Consequently, risk assessments based solely on as‐synthesized ZnO NPs may be insufficient [12, 13, 14, 15, 16, 17]. Zhang et al. pointed out that with the development of facet engineering, nanomaterials (including ZnO NPs) with distinct primary exposed facets were gradually used in food packages and additives, resulting in their interactions with saliva. Their findings demonstrated the facet‐dependent transformation and toxicity of ZnO NPs, showing that ZnO nanoneedles compared to ZnO nanoflakes would likely transform into amorphous Zn_3_(PO_4_)_2_ in the synthetic saliva and possess strong toxicity to Caco‐2 cells [18]. Hatch et al. also demonstrated that inhaled ZnO NPs, including those from commercial products, undergo rapid and extensive chemical transformation within 24 h of incubation in simulated lung fluid (SLF), a process driven by the fluid's pH and chemical complexity [7]. These findings underscore that even during short‐term exposure, ZnO NPs in consumer products can experience substantial transformation, highlighting the need for incorporating transformed NPs in toxicity evaluations. Our previous study revealed that ZnO NPs aged in pure water for a certain period (20–120 days) underwent hydrolysis, releasing Zn^2+^ ions, while partially transforming into the solid phase zinc hydroxide carbonate (Zn_5_(CO_3_)_2_(OH)_6_) [19]. Subsequent cellular experiments and follow‐up studies in nude mice demonstrated that aged NPs exhibited enhanced genotoxicity (DNA damage and gene mutations) as well as an increased ability to induce cell malignant transformation and tumor formation in nude mice, suggesting their potential role as key drivers in cancer initiation and progression [19, 20]. However, research on the carcinogenicity of respiratory exposure to ZnO NPs has so far primarily focused on whether pristine/fresh NPs can induce primary lung cancer, with predominantly negative outcomes (e.g., confirming only systemic or local inflammation and fibrosis) [21, 22, 23]. Studies investigating the carcinogenic and cancer‐promoting potential of aged ZnO NPs remain remarkably scarce.

Tumor microenvironment (TME) is a highly dynamic and heterogenous ecosystem composed of tumor cells, stromal cells (such as cancer‐associated fibroblasts [CAFs] and endothelial cells), immune cells (including tumor‐associated macrophages [TAMs] and T cells), extracellular matrix (ECM), vasculature, and diverse signaling molecules [24]. In contrast to earlier perspectives that viewed tumors primarily as masses of malignant cells, current research emphasizes the TME as a pivotal regulator of tumorigenesis, progression, and therapeutic resistance [25]. CAFs are one of the most abundant stromal components in the TME. They secrete growth factors (TGF‐β, FGF and HGF) and remodel the ECM through matrix metalloproteinases (MMPs), thereby promoting invasion, angiogenesis, and immune evasion [26]. Similarly, TAMs, especially M2‐like polarized subsets, enhance immunosuppression, metastasis, and therapy resistance via the production of IL‐10, VEGF, and arginase‐1 [27]. TME also fosters immunosuppression through recruitment of regulatory T cells (Tregs), myeloid‐derived suppressor cells (MDSCs), and exhausted T cells expressing immune checkpoints such as PD‐1 and CTLA‐4 [28]. Furthermore, ECM stiffness and fibrotic barriers impair T‐cell infiltration, leading to immune exclusion [29]. Given its central role in malignancy, TME has emerged as a promising therapeutic target. Immune checkpoint inhibitors (anti‐PD‐1/PD‐L1, anti‐CTLA‐4) aim to reinvigorate T‐cell exhaustion [30]. Strategies targeting CAFs‐ such as FAP‐directed CAR‐T cells or TGF‐β inhibitors‐ aim to alleviate desmoplasia and enhance drug penetration [31]. Despite these advances, TME heterogeneity, adaptive resistance, and compensatory signaling pose ongoing challenges. Emerging tools like single‐cell sequencing, spatial transcriptomics, and patient‐derived organoids are providing unprecedented insights into TME plasticity and guiding the development of rational combination therapies [32].

The CXCL10/CXCR3 axis critically regulates tumor progression, immune evasion, and metastatic spread in numerous cancers. CXCL10 (also known as IP‐10) binds to its receptor CXCR3, a G protein‐coupled receptor expressed on immune cells, endothelial cells, and cancer cells [33]. It has been reported that CXCL10/CXCR3 axis directly mediated tumor cell motility and invasion to enhance metastasis [34, 35]. Beyond cell‐autonomous effects, this axis orchestrates immunosuppressive microenvironments conducive to metastasis. For example, CXCL10 promotes the accumulation of monocyte‐like myeloid‐derived suppressor cells by activating p38 MAPK signaling under tumor conditions [36]; CXCL10 conditions alveolar macrophages (Mφ) within the premetastatic niche initiates recruitment of monocytic MDSCs (mo‐MDSCs) and promote lung metastasis by increasing CCL12 expression [37]. Clinically, elevated serum CXCL10 correlates with metastatic progression and poor prognosis in multiple cancer types, highlighting its diagnostic and prognostic value [38]. However, context‐dependent functions of CXCL10 complicate therapeutic targeting, necessitating precise patient stratification [33].

Given the inhalation risk of aerosolized ZnO NPs from nano‐enabled products and their potential aging in air, this study utilized an intratracheal instillation mouse model combined with tail vein injection of breast cancer cells to evaluate the metastatic potential of fresh versus aged ZnO NPs. Compared to fresh ZnO NPs, aged NPs induced markedly greater metastatic burden by fostering an immunosuppressive and fibrotic TME. This process was driven by ZnO NP‐induced CXCL10 upregulation, which promoted M2 macrophage polarization and MDSCs recruitment, activated CAFs, and stimulated Wnt/β‐catenin signaling in tumor cells. Our results reveal a CXCL10‐mediated mechanism underlying aged ZnO NP‐aggravated breast cancer metastasis and provide critical evidence for refining nanomaterial risk assessment.

## 2 Results

### 2.1 Breast Cancer Fosters Fibrotic, Immunosuppressed Lung Niches

Breast cancer fosters an immunosuppressive and fibrotic microenvironment in the lung that facilitates metastatic colonization [39]. To delineate the specific features of this pulmonary niche, we conducted a comprehensive single‐cell RNA sequencing analysis of murine lung tissues with (GSM7518067, GSM7518068, GSM7518069) or without (GSM6687572) breast cancer lung metastasis. After quality control filtering, cells were categorized into 10 distinct lineages, including endothelial cells (Cdh5, Pecam1), myeloid cells (Cd68, Alf1, S100a8), T_NK cells (Cd3d, Nkg7), B cells (Cd79a, Cd79b), fibroblasts (Col1a1, Col1a2), epithelial cells (Krt18, Krt8), pericyte cells (Vtn, Ptgfrb), SMC cells (Acta2, Mustn1), proliferating cells (Mki67, Top2a) and basophil cells (Ms4a2, Cpa3), based on expressions of cell‐type specific markers (Figure S1A, B). The proportion and cell numbers of each cell type were shown in (Figure S1C, D). Here, we placed emphasis on myeloid cells and fibroblasts based on prior evidence [40, 41]. Re‐clustering of myeloid cells revealed six subpopulations (Figure S2A, B). Significantly expanded M2‐like macrophages and MDSCs were observed in metastatic lung tissue (Figure S2C, D), accompanied by activation of pathways supporting an immunosuppressive niche (Figure S2E, F). Moreover, fibroblasts were subdivided into three clusters, including α‐SMA^+^ and Cd140a^+^ CAFs subsets according to the expressions of two classical CAFs markers: α‐SMA, Cd140a (Figure S2G, H), both of which were enriched in metastatic lungs (Figure S2I, J). These CAFs exhibited upregulated activation pathways indicative of a pro‐fibrotic and tumor‐supportive phenotype (Figure S2K, L). Collectively, these findings illustrate that breast cancer metastasis reprograms the lung into an immunosuppressive and fibrotic niche, dominated by specific myeloid and CAF subpopulations that support metastatic growth.

### 2.2 ZnO NPs Drive Breast Cancer Lung Metastasis

This study investigates whether inhaled ZnO NPs influence cancer progression through systemic mechanisms beyond local pulmonary inflammation [42, 43] or direct neoplastic effects [44, 45]. More importantly, our objective is to elucidate the oncogenic effects of aged ZnO NPs, particularly given the reality of human exposure to aged/transformed NPs and the significantly enhanced mutagenic potential inherent in aged NPs [19]. TEM images revealed a marked morphological transformation in our NPs, shifting from predominantly regular crystalline structures to predominantly large, irregular amorphous phases (Figure 1A). XRD diffraction patterns of aged ZnO NPs confirmed the partially conversion of ZnO to sheet‐like hydrozincite (Zn_5_(CO_3_)_2_(OH)_6_) (Figure 1B). The details of the physicochemical transformation process have been comprehensively analyzed in our previous work [19]. The experimental timeline included NP administration, establishment of 4T1‐lucferase allografts, and metastasis monitoring (Figure 1C). Bioluminescence imaging revealed robust lung metastasis in mice exposed to fresh ZnO NPs, with further augmentation in aged NP groups compared to PBS controls (Figure 1D–G). In addition, 4T1‐luc cells in fresh ZnO NP‐exposed mice induced larger and more numerous metastatic lesions observed in H&E staining sections (Figure 1H,I), and these lung metastatic lesions showed a higher percentage of Ki‐67 positive signals (Figure 1J, K), indicating enhanced cell proliferation compared with PBS control group. Histopathological quantification confirmed significantly greater upregulation of all evaluated indices in aged ZnO NP‐exposed mice (Figure 1H–K). Furthermore, survival period was significantly reduced in fresh ZnO NP‐treated mice and further decreased following aged NPs exposure (Figure 1L). Together, these findings demonstrate that pulmonary ZnO NPs exposure, particularly aged NPs, systemically could promote breast cancer lung metastasis and accelerate mortality.

**FIGURE 1:**
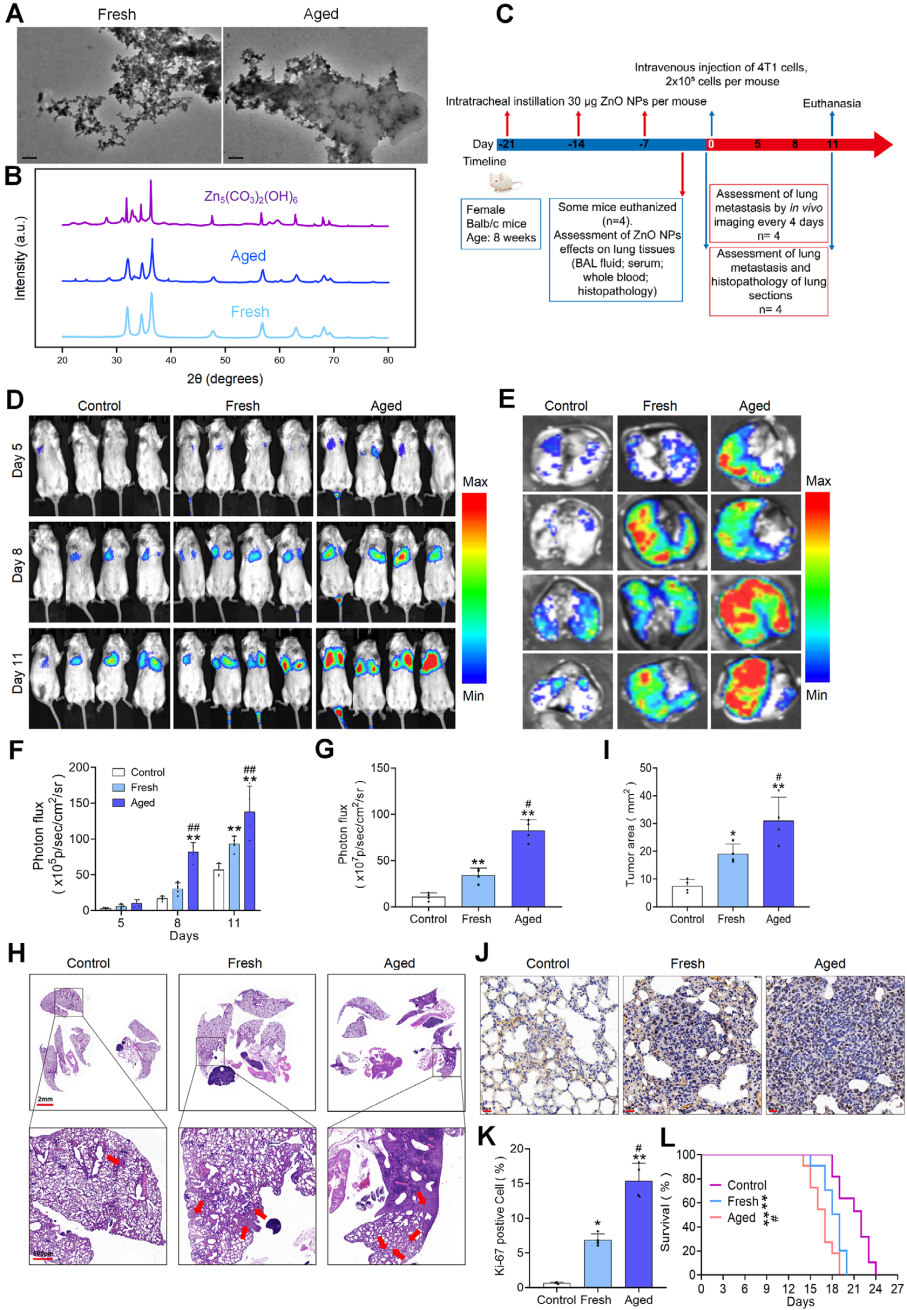
Pulmonary exposure of ZnO NPs promotes lung metastasis in breast cancer. (A) TEM images of fresh and aged NPs at 100 mg mL^−1^ in Milli‐Q water. Scale bar: 0.2 µm. (B) XRD patterns of Zn_5_(CO_3_)_2_(OH)_6_ reference, fresh, and aged NPs. (C) Experimental timeline: mice received three weekly intratracheal instillations of PBS, fresh, or aged ZnO NPs, followed by intravenous injection of 4T1‐luc breast cancer cells. (D–G) Longitudinal in vivo (D) and ex vivo (E) bioluminescence imaging with corresponding quantitative analyses (F, G) showing metastatic burden in Control, fresh, or aged ZnO NP‐exposed groups (*n* = 4). (H, I) H&E staining (H) and quantification (I) of metastatic lung lesions in the indicated groups (*n* = 4). Scale bar: 2 mm (top) and 500 µm (bottom). (J, K) Ki‐67 immunohistochemistry (J) and quantification (K) indicating proliferation rate in Control, fresh, or aged ZnO NP‐exposed metastases (*n* = 4). Scale bar: 20 µm. (L) Kaplan‐Meier survival curves of mice in the indicated groups followed by intravenous injection of 4T1‐luc cells (*n* = 10). All data were represented as the mean ± SD. P values were calculated by Kaplan−Meier analysis in L, others with one‐way ANOVA followed by Tukey's test. ^*^p < 0.05, ^**^ p < 0.01 vs control group; *^#^ p* < 0.05, *^##^ p* < 0.01 vs fresh NPs group.

### 2.3 Pulmonary Niche Remodeling by ZnO NPs Exposure

To uncover the cellular and molecular mechanisms by which ZnO NPs enhanced breast cancer metastasis, lung tissues in each group were harvest for scRNA‐seq, flow cytometry, histological and molecular analyses (Figure S3A). scRNA‐seq identified 8 major cell types, including T_NK cells (Cd3e, Nkg7, Cd3d), myeloid cells (Cd68, Csf1r, S100a8, S100a9), endothelial cells (Cd79b, Cd79a), fibroblasts (Cdh5, Ramp2), AT2 cells (Col3a1, Dcn), B cells (Sftpd, Sftpa1), Club (Scgb3a2), epithelial cells (Epcam, Krt8), based on canonical markers in both aged ZnO NPs and PBS control groups (Figure S3B, C). While no significant differences were observed in either the absolute counts or proportional distribution of myeloid cells and fibroblasts between aged ZnO NPs and PBS control groups (Figure S3D, E), we consequently performed in‐depth characterization of subtype‐specific variations within these two cellular populations.

Myeloid cells were subdivided into six distinct subsets (Figure 2A) after re‐clustering based on the cell‐type specific markers (Figure 2B). Notably, aged ZnO NPs exposure significantly increased the abundance and proportion of M2 macrophages and MDSCs (Figure 2C, D), and upregulated pathways supporting their immunosuppressive functions (Figure 2E, F). To validate the scRNA‐seq results, histological analysis revealed pronounced peribronchial immune infiltration in ZnO NP‐exposed lungs, which was more severe in aged NP‐treated mice (Figure S4A). Immunohistochemistry (IHC) analysis confirmed that fresh ZnO NPs exposure clearly increased, and aged ZnO NPs increased much more the expression of inflammatory biomarkers: F4/80 and COX‐2 in the lung tissues (Figure S4B, C). Moreover, the mRNA levels of inflammatory genes such as S100A8, S100A9, TIMP1, CXCL1, CXCL9, IL10, COX2, CD206, ARG1, Fizz1 and PPARγ increased in the lung tissue and bronchoalveolar lavage fluid (BALF)‐derived macrophages of fresh ZnO NP‐exposed mice, with highest expression following aged NPs exposure (Figure S4D, E). Furthermore, we also examined both monocytic Ly6G^low^Ly6C^+^ and granulocytic Ly6G^+^Ly6C^low^ MDSCs in spleens and lungs using flow cytometry and found increased granulocytic MDSCs accumulation in aged ZnO NP‐exposed mice (Figure S4F–I). Consistent with an M2‐skewed response, fresh ZnO NPs enhanced CD206^+^ macrophage infiltration, whilst aged ZnO NPs further amplified this effect (Figure S4J–M).

**FIGURE 2:**
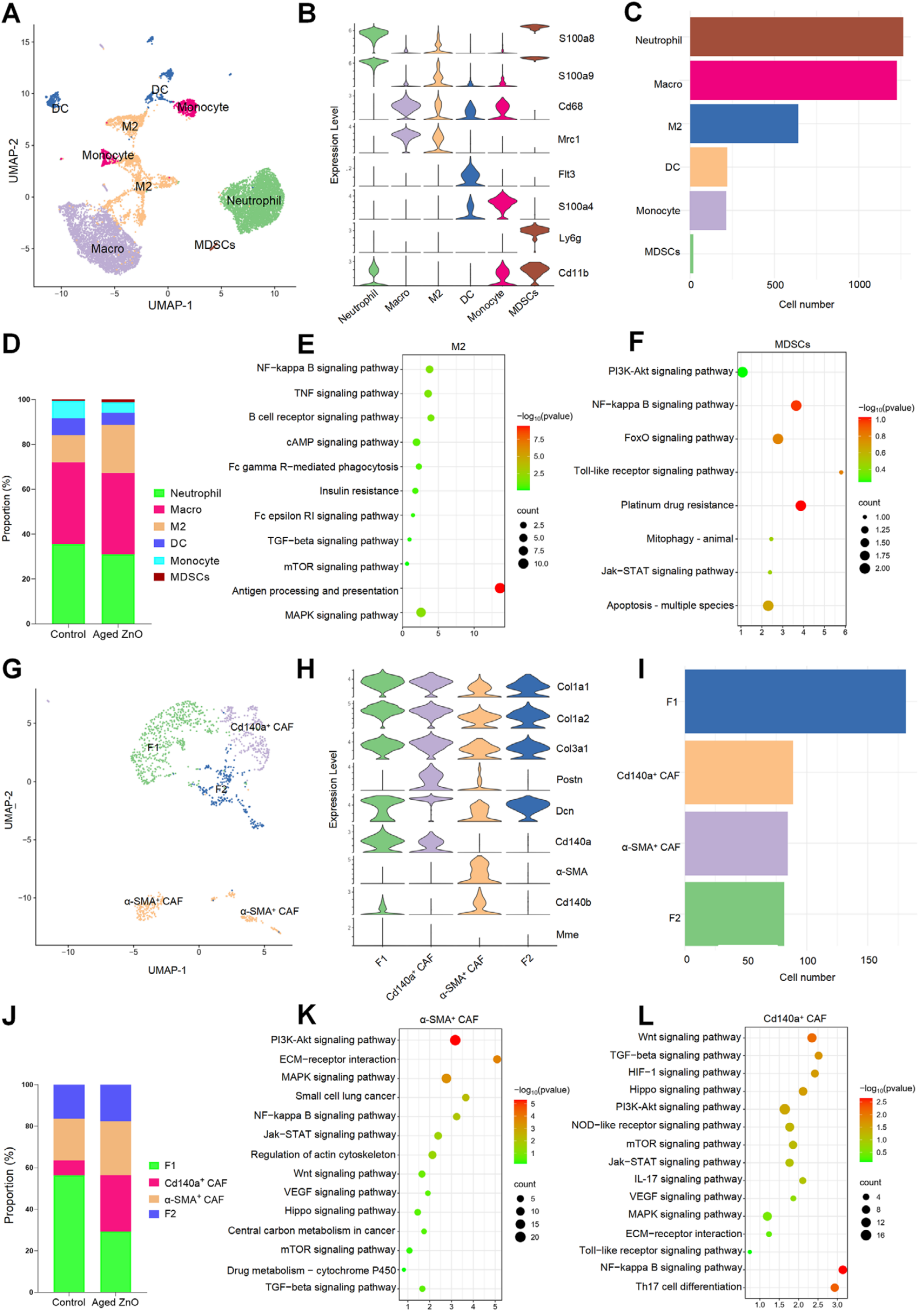
Aged ZnO NPs remodel the pulmonary metastatic niche through expansion of pro‐tumorigenic stromal populations. (A) UMAP clustering analysis of myeloid subsets in the lung tissues derived from 4T1‐luc tumor‐bearing mice exposed to aged ZnO NPs or control. (B) Violin plot showing relative expression of select marker genes for each cell type shown in (A). (C) Bar plot showing total cell numbers in each cell type shown in (A). (D) Comparison of myeloid subsets proportions in lung tissues derived from 4T1‐luc tumor‐bearing mice exposed to aged ZnO NPs versus control. (E, F) KEGG pathway enrichment analysis of M2 macrophages (E) and MDSCs (F) in lung tissues upon aged ZnO NPs exposure. (G–I) UMAP clustering (G), marker expression (H), and counts (I) of fibroblast subsets in the lung tissues derived from 4T1‐luc tumor‐bearing mice exposed to aged ZnO NPs or control. (J) Comparison of fibroblasts subsets proportions in lung tissues derived from 4T1‐luc tumor‐bearing mice exposed to aged ZnO NPs versus control. (K, L) KEGG pathway enrichment analysis of α‐SMA^+^ (K) and Cd140a^+^ (L) CAFs in lung tissues derived from 4T1‐luc tumor‐bearing mice exposed to aged ZnO NPs.

Fibroblasts were re‐clustered into four subgroups according to the expressions of two classical CAFs markers: α‐SMA, Cd140a (Figure 2G, H). Both α‐SMA^+^ and Cd140a^+^ CAFs were notably expanded in aged NP‐exposed lungs (Figure 2I, J), and exhibited activation of pro‐fibrotic signaling pathways (Figure 2K, L). Increased collagen deposition was observed via Masson's trichrome staining in fresh ZnO NP‐exposed mice, with the most severe fibrosis in aged ZnO NPs group (Figure S5A). IHC analysis revealed that exposure to fresh ZnO NPs markedly upregulated the expression of fibrotic biomarkers (FN1, FSP1 and bFGF) in the lung tissues, with aged ZnO NPs inducing even more pronounced upregulation (Figure S5B, C). Consistent with these findings, mRNA levels of fibrosis‐associated genes (FN1, FSP1, bFGF, VEGFA, α‐SMA, and Cd140a) were elevated in both whole lung tissue and isolated fibroblasts from fresh ZnO NP‐exposed mice, and were further enhanced following exposure to aged ZnO NPs (Figure S5D, E). Flow cytometry and immunofluorescence validated the expansion of Cd140a^+^ and α‐SMA^+^ CAFs following fresh ZnO NPs challenge, with maximal effects in aged ZnO NPs group (Figure S5F–I). Taken together, ZnO NPs, particularly aged NPs, drive the establishment of an immunosuppressive and fibrotic microenvironment conducive to breast cancer metastasis.

### 2.4 Mφ and Fibroblasts Re‐Educated by ZnO NPs Promote Metastasis

Given the critical involvement of macrophages and fibroblasts in regulating breast cancer metastasis, we established a co‐culture system to directly evaluate the influence of ZnO NP‐educated macrophages/fibroblasts on metastatic behavior (Figure 3A). Transwell assays demonstrated that murine 4T1 cells co‐cultured with RAW264.7 macrophages pre‐treated with fresh ZnO NPs exhibited enhanced migration and invasion, with this effect being markedly more pronounced when aged ZnO NPs were employed (Figure S6A–C). Similarly, human breast cancer cells (MCF‐7 and MDA‐MB‐231) displayed increased motility when co‐cultured with THP‐1‐derived macrophages exposed to fresh or aged ZnO NPs (Figure S6D–I). Primary macrophages isolated from bronchoalveolar lavage fluid of fresh/aged ZnO NP‐exposed mice also enhanced migration and invasive capacity of 4T1‐luc cells in ex vivo assays (Figure S6J–L). To assess functional metastasis, 4T1‐luc cells pre‐conditioned with fresh/aged ZnO NP‐treated RAW264.7 macrophages were injected into mice via tail vein. Bioluminescence imaging revealed significantly greater pulmonary metastatic burden in mice receiving cells stimulated with fresh or aged ZnO NPs compared to PBS controls, with the most severe metastasis following aged ZnO NPs exposure (Figure 3B–D). These mice also exhibited markedly reduced survival (Figure 3E). Moreover, fresh/aged ZnO NP‐stimulated 4T1‐luc cells induced larger and more numerous metastatic lesions observed in H&E staining sections, and these lung metastatic lesions showed a higher percentage of Ki‐67 positive signals, indicating enhanced cell proliferation compared with the PBS control group (Figure 3F–H).

**FIGURE 3:**
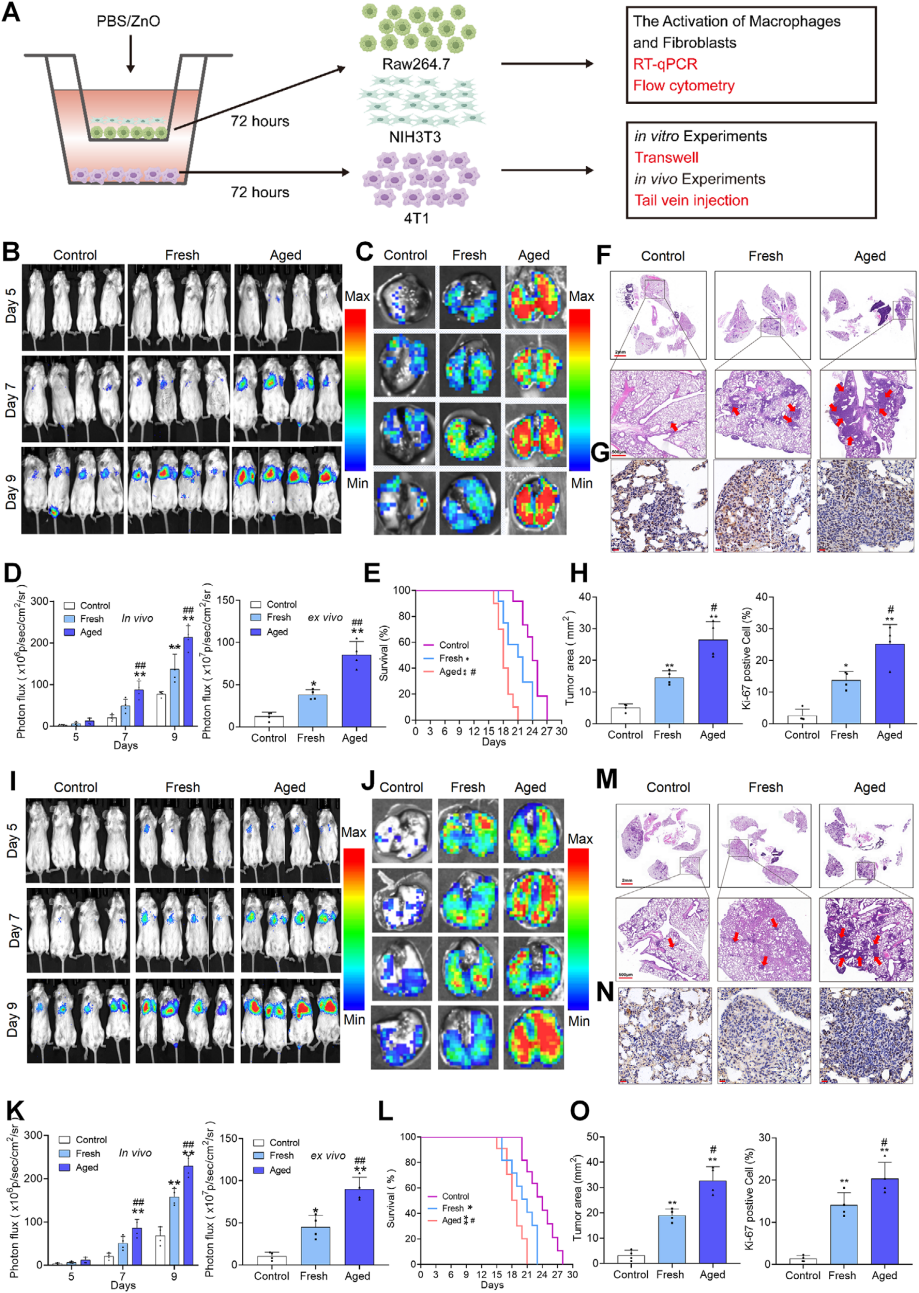
Aged ZnO NP‐primed macrophages and fibroblasts drive breast cancer metastasis. (A) Experimental design. NIH‐3T3 or Raw264.7 cells were stimulated with control, fresh ZnO or aged ZnO NPs (7.5 µg/mL), co‐cultured with 4T1 cells, and subsequently harvested for downstream assays. (B–D) Lung metastatic burden of macrophage‐primed 4T1 cells derived from the indicated groups, as assessed by longitudinal (B) and ex vivo (C) bioluminescence with quantification (D) (*n* = 4). (E) Kaplan‐Meier survival analysis of mice injected with Raw264.7‐stimulated 4T1‐luc cells of each group (*n* = 10). (F) Representative H&E staining of lung metastases. Scale bar: 2 mm (top) and 500 µm (bottom). (G) Representative Ki‐67 immunohistochemistry (*n* = 4). Scale bar: 20 µm. (H) Quantification of metastatic area and Ki‐67‐positive cells in lung sections (*n* = 4). (I‐K) In vivo (I), ex vivo (J) bioluminescence imaging and quantification (K) of lung metastases formed by indicated NIH‐3T3‐primed 4T1 cells at endpoint (*n* = 4). (L) Kaplan–Meier survival analysis of mice injected with 4T1‐luc cells co‐cultured with indicated NIH‐3T3 cells (*n* = 10). (M) Representative H&E staining of lung metastatic foci (*n* = 4). Scale bar: 2 mm (top) and 500 µm (bottom). (N) Representative IHC staining of Ki‐67 (*n* = 4). Scale bar: 20 µm. (O) Quantification of metastatic area and Ki‐67–positive cells (*n* = 10). All data were represented as the mean ± SD. P values were calculated by Kaplan‐Meier analysis in E and L, others with one‐way ANOVA followed by Tukey's test. ^*^p < 0.05, ^**^p < 0.01 vs control group; *^#^p* < 0.05, *^##^p* < 0.01 vs fresh NPs group.

Parallel experiments using fibroblasts yielded consistent results: NIH3T3 pre‐treated with fresh ZnO NPs enhanced the migratory and invasive capacities of murine 4T1 cells, effects that were further potentiated by aged ZnO NPs (Figure S6M–O). Further co‐culturing human mammary carcinoma cells (MCF‐7 and MDA‐MB‐231) with human fibroblasts IMR‐90 treated with fresh/aged ZnO NPs unanimously displayed elevated migration and invasion (Figure S6P–U). To determine functional metastatic potential in vivo, we intravenously injected 4T1‐luc cells that had been pre‐conditioned with NIH3T3 fibroblasts exposed to fresh ZnO NPs, aged ZnO NPs or PBS. Bioluminescence imaging revealed a significant increase in pulmonary metastasis burden in mice receiving cells stimulated with either fresh or aged ZnO NPs compared to the PBS control group (Figure 3I–K). Furthermore, mice injected with fresh/aged ZnO NP‐stimulated 4T1‐luc cells showed a significantly lower survival rate compared with PBS control mice (Figure 3L). Histological evaluation confirmed the presence of larger and more numerous metastatic lesions in the lungs, which displayed elevated Ki‐67^+^ proliferation rates, indicating enhanced tumor growth following exposure to ZnO NP‐educated fibroblasts (Figure 3M–O).

We further focused on the function variations of macrophages/fibroblasts/MDSCs based on the co‐culture system. Fresh ZnO NPs exposure induced M2‐like polarization in macrophages, marked by increased CD206^+^ protein expression (Figure S7A–D) and mRNA upregulation of CD206 and ARG1 (Figure S7E), with aged ZnO NPs inducing even more pronounced upregulation (Figure S7A–E). Fibroblasts adopted a CAF‐like phenotype, showing increased abundance of Cd140a^+^/α‐SMA^+^ cells (Figure S7F–I) and elevated expression of FN1, FSP1, α‐SMA, and Cd140a (Figure S7J). Additionally, when 4T1‐luc cells were co‐cultured with primary mouse myeloid cells (Figure S7K), exposure to fresh ZnO NPs significantly expanded granulocytic Ly6G^+^Ly6C^low^ MDSCs population, an effect that was further enhanced by aged ZnO NPs (Figure S7L, M).

Collectively, our data indicated that aged ZnO NPs exhibit a heightened capacity to drive breast cancer metastasis by reprogramming stromal components including induction of M2 macrophages, activation of CAFs, and expansion of MDSCs.

### 2.5 ZnO NPs Induce CXCL10 Upregulation in Lung Mφ and Fibroblast

To further explore the underlying molecular mechanisms by which ZnO NPs remodel the pulmonary TME, we profiled inflammatory cytokines and chemokines in BALF and serum from the mice exposed to fresh, aged ZnO NPs or PBS‐with or without tumor cell injection using a magnetic Luminex assay (Figure 4A). We identified all inflammation‐associated cytokines/chemokines upregulated under these conditions (Figure 4B–E), revealing that CXCL10 and CXCL13 were commonly elevated across all four experimental groups (Figure 4F). Notably, CXCL10 was persistently induced by fresh ZnO NPs and was further enhanced by aged ZnO NPs, as validated by Enzyme‐Linked Immunosorbent Assay (ELISA) (Figure 4G). Consistent with these findings, CXCL10 mRNA levels were increased in lung tissues, primary pulmonary fibroblasts, and macrophages from fresh ZnO NP‐exposed mice, with the highest expression observed following aged ZnO NPs treatment (Figure 4H). Further analysis of our single‐cell RNA‐seq data confirmed that CXCL10 is primarily expressed in fibroblasts and myeloid cells (Figure 4I, J), with particularly prominent expression in macrophages within the myeloid compartment (most notably the M2‐polarized subset) (Figure 4K, L). Although we also detected substantial CXCL10 expression in dendritic cells (DCs), this observation falls outside the primary scope of the current study and will not be further addressed herein. This indicated that pulmonary fibroblasts and macrophages could be the major sources of ZnO NP‐induced CXCL10. In addition, functional assays confirmed that rCXCL10 significantly enhanced the migration and invasion capacity of 4T1‐luc cells (Figure 4M, N). Taken together, these results demonstrate that ZnO NPs exposure triggers CXCL10 secretion from pulmonary fibroblasts and macrophages, thereby facilitating breast cancer metastasis.

**FIGURE 4:**
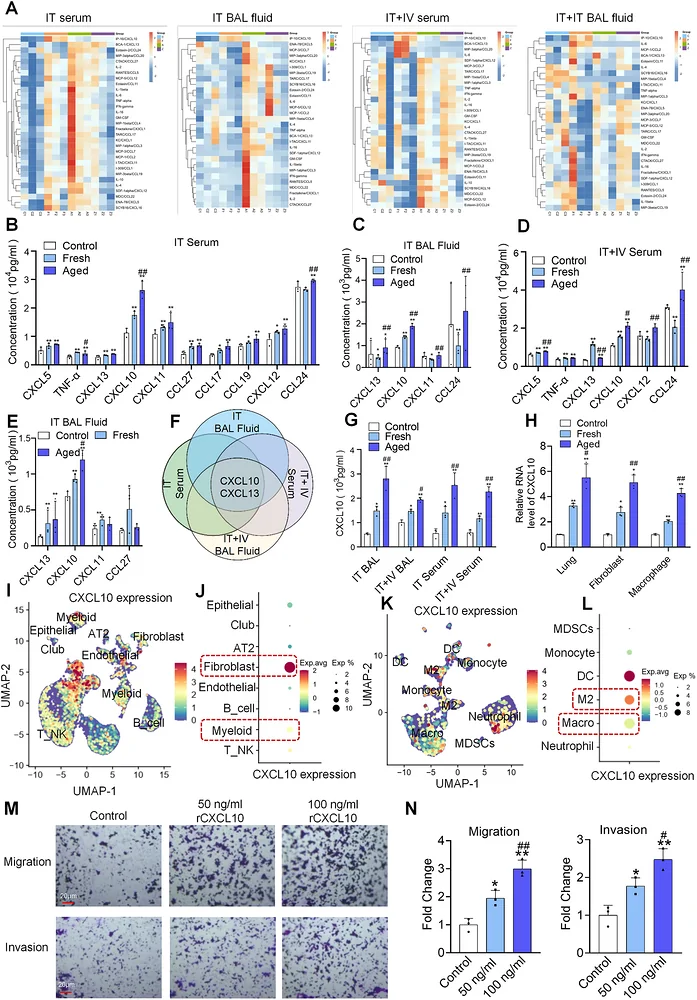
CXCL10 is upregulated in both macrophages and fibroblasts responding to aged ZnO NPs exposure. (A) Heatmaps exhibited multiplex cytokine profiling of serum and BALF from IT (PBS/fresh NPs/aged NPs intratracheal instillation) and IT + IV (IT exposure followed by intravenous injection of 4T1 cells) models (*n* = 3). (B–E) Concentrations of significantly dyregulated cytokines in serum and BALF from mice exposed to ZnO NPs (fresh or aged), with or without subsequent 4T1 cell injection (*n* = 3). (F) Venn plot showed the overlapping cytokines in serum and BALF between IT and IT + IV models. (G) ELISA detection of CXCL10 concentrations in serum and BALF of IT and IT + IV groups (*n* = 3). (H) qPCR analysis of CXCL10 mRNA in lung tissue, primary fibroblasts, and alveolar macrophages post‐IT, normalized to GAPDH (*n* = 3). (I, J) UMAP visualization (I) and dot plot (J) displaying CXCL10 expression across annotated cell types. (K, L) UMAP visualization (K) and dot plot (L) displaying CXCL10 expression across myeloid cell types. (M) Representative images of invasion and migration assays of 4T1 cells stimulated with 50 or 100 ng/mL murine recombinant CXCL10 (rCXCL10) for 72 h (*n* = 3). (N) Quantification of invasion and migration assays (*n* = 3). All data were represented as the mean ± SD. P values were calculated by one‐way ANOVA followed by Tukey's test. ^*^p < 0.05, ^**^p < 0.01 vs control group; *^#^p* < 0.05, *^##^p* < 0.01 vs fresh NPs group.

### 2.6 CXCL10 Loss Prevents ZnO NP‐Induced Immunosuppressive TME

To further characterize whether CXCL10 mediates ZnO NP‐driven remodeling of the pulmonary TME, we co‐cultured 4T1‐luc cells with CXCL10‐depleting RAW264.7 macrophages or NIH3T3 fibroblasts prior to fresh or aged ZnO NPs treatment (Figure 5A). Endogenous CXCL10 in RAW264.7 cells was markedly silenced by transfection of a siRNA pool against CXCL10 (Figure 5B). Depletion of CXCL10 markedly attenuated the upregulation of M2 macrophage markers (CD206, ARG1) induced by fresh/aged ZnO NPs (Figure 5C), and reduced the proportion of CD206^+^ macrophages (Figure 5D, E). Similarly, after confirming the knockdown efficiency of CXCL10 in NIH3T3 cells (Figure 5F), we found that CXCL10 knockdown reversed the fresh/aged ZnO NP‐induced increase in Cd140a^+^ fibroblasts abundance (Figure 5G, H) and decreased the upregulated expression of CAF markers including FN1, FSP1, α‐SMA, and Cd140a (Figure 5I). Furthermore, co‐culturing 4T1‐luc cells with mouse primary myeloid cells transfected with an siRNA pool against CXCL10 (Figure 5J, K) revealed that CXCL10 suppression significantly reduced the expansion of granulocytic MDSCs (Ly6G^+^Ly6Cˡ°ʷ) induced by fresh/aged ZnO NPs (Figure 5L, M). Collectively, our data demonstrate that CXCL10 is essential for ZnO NP‐induced accumulation of immunosuppressive and pro‐fibrotic stromal cells.

**FIGURE 5:**
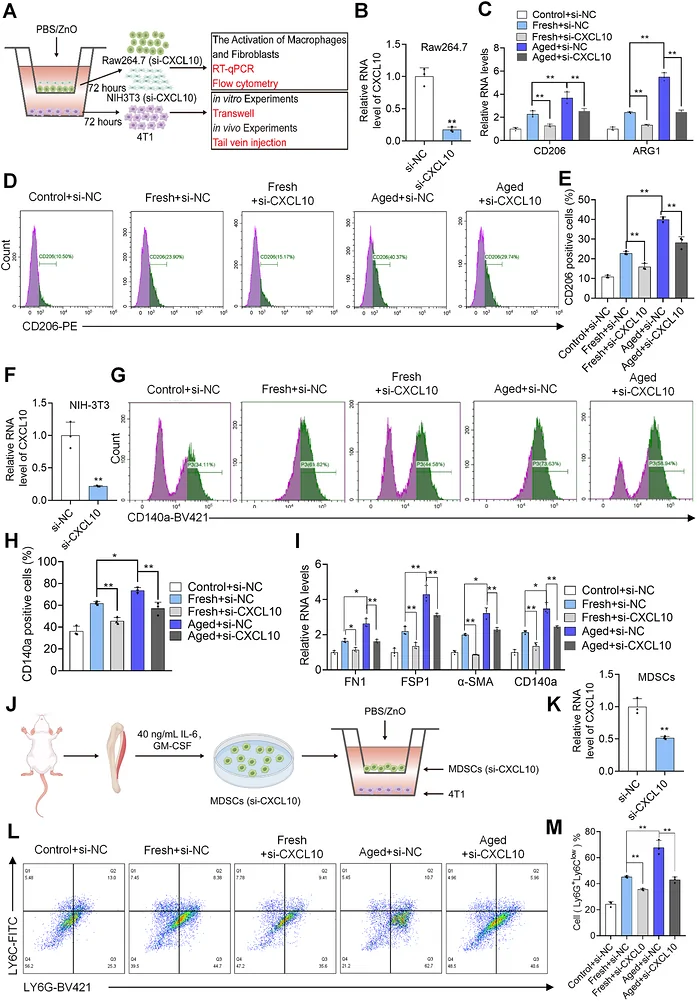
CXCL10 silencing abrogates aged ZnO NPs–induced accumulation of pro‐metastatic stromal populations. (A) Schematic of coculture model: NIH‐3T3 or Raw264.7 cells with CXCL10 knockdown were stimulated with PBS, fresh or aged NPs (7.5 µg/mL), co‐cultured with 4T1 cells for 72 h. (B) qPCR analysis confirming CXCL10 knockdown in Raw264.7 cells (*n* = 3). (C‐E) CXCL10 knockdown in Raw264.7 macrophages attenuates ZnO NP‐enhanced M2 polarization, as shown by reduced Arg1, Mrc1 expression (C) and CD206^+^ cell frequency (D, E) (*n* = 3). (F) qPCR analysis confirming CXCL10 knockdown in NIH‐3T3 cells (*n* = 3). (G, H) Flow cytometric analysis (G) and quantification (H) of the percentage of CD140a‐positive cells among CXCL10‐knockdown NIH‐3T3 cells in the co‐culture system (*n* = 3). (I) qPCR analysis of CAF‐related genes (FN1, FSP1, α‐SMA, CD140a) in CXCL10‐knockdown NIH‐3T3 cells from the co‐culture model (*n* = 3). (J) Schematic illustration of primary MDSCs preparation: primary mouse bone marrow‐derived macrophages were differentiated into MDSCs by supplement of GM‐CSF (40 ng/mL) and IL‐6 (40 ng/mL). MDSCs with CXCL10 knockdown were stimulated with PBS, fresh ZnO NPs, or aged ZnO NPs (7.5 µg/mL), then co‐cultured with 4T1 cells for 72 h. Cells were collected for further experiments. (K) qPCR analysis of relative CXCL10 mRNA expression in MDSCs with CXCL10 knockdown (*n* = 3). (L, M) Flow cytometric analysis (L) and quantification (M) of the proportion of granulocytic CD11b^+^Ly6G^+^Ly6C^low^ PMN‐MDSCs in the co‐culture system after CXCL10 knockdown (*n* = 3). All data were represented as the mean ± SD. P values were calculated by one‐way ANOVA followed by Tukey's test. ^*^ *p*<0.05; ^**^ *p*<0.01.

We next elucidated the molecular mechanism through which CXCL10 regulated metastasis. RNA‐seq of CXCL10‐treated 4T1‐luc cells revealed significant transcriptomic alterations, with PCA confirming high reproducibility across samples (Figure S8A). The volcano plot displayed differentially expressed genes (Figure S8B). KEGG pathway enrichment identified Wnt signaling as the top pathway affected by CXCL10 (Figure S8C). Validation experiments confirmed that CXCL10 upregulated Wnt‐responsive genes (c‐MYC, CyclinD1, Survivin) at both RNA (Figure S8D, E) and protein (Figure S8F–H) levels. Moreover, CXCL10 increased the protein levels of main regulators of Wnt signaling such as total β‐catenin and phosphorylated GSK3β (Figure S8I–K). Except for β‐catenin's protein expression levels, its subcellular localization was also crucial for assessing the activity of Wnt pathway. Immunofluorescence analysis further demonstrated enhanced nuclear translocation of β‐catenin in both 4T1 and MCF‐7 cells following CXCL10 treatment (Figure S8L). Furthermore, in vitro functional assays revealed that the promigratory and proinvasive effects of CXCL10 on breast cancer cells were markedly suppressed following pharmacological blockade of the Wnt signaling pathway (Figure S9). These findings indicate that CXCL10 could drive breast cancer metastasis by activating Wnt/β‐catenin pathway.

### 2.7 Blocking CXCL10/CXCR3 Axis Inhibits ZnO NP‐Driven Metastasis

To determine whether CXCL10 mediates the pro‐metastatic effects of ZnO NPs, we treated 4T1‐luc tumor‐bearing mice with the selective CXCR3 antagonist AMG487 following exposure to fresh or aged ZnO NPs (Figure 6A). Consistent with prior observations, both fresh and aged ZnO NPs significantly enhanced lung metastasis and increased Ki‐67^+^ proliferating cells in mice receiving tail‐vein‐injected 4T1‐luc cells, with maximal effects in the aged ZnO NPs group. Notably, AMG487 treatment completely reversed these effects, as evidenced by bioluminescence imaging (Figure 6B–E), histopathological (Figure 6F–I) and survival analysis (Figure 6J). Taken together, these findings demonstrate that the metastasis‐promoting effects of ZnO NPs are critically dependent on CXCL10/CXCR3 signaling pathway.

**FIGURE 6:**
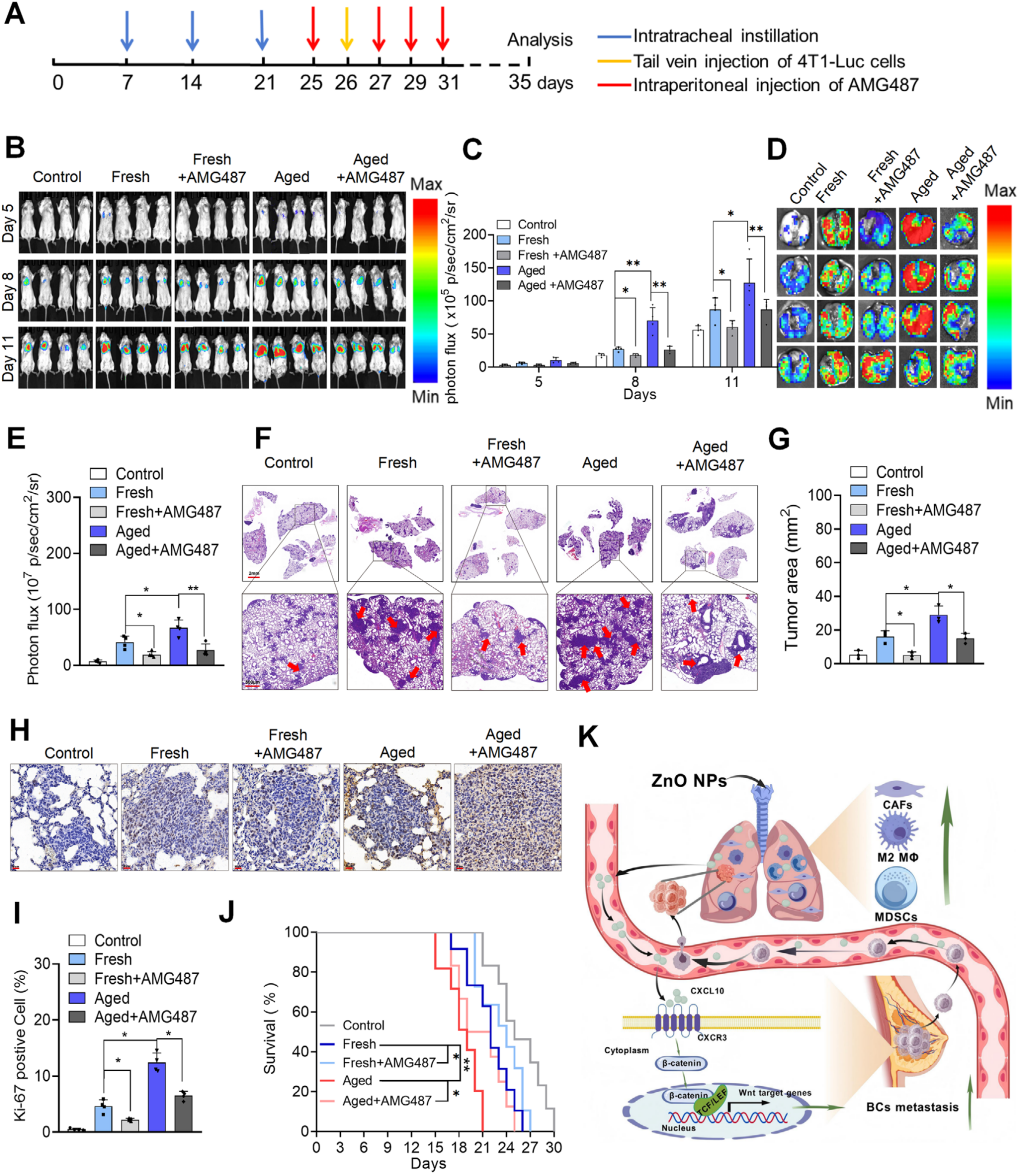
Pharmacological blockade of CXCL10/CXCR3 abrogates ZnO NP–enhanced metastasis. (A) Timeline of intraperitoneal administration of the CXCL10 receptor antagonist AMG487, ZnO NPs intratracheal instillation, and injection of 4T1‐Luc cells. (B–I) AMG487 treatment significantly reduced ZnO NP–induced lung metastatic burden, as shown by longitudinal and ex vivo bioluminescence (B–E), with corresponding decreases in lesion area (F, G) and tumor cell proliferation (Ki‐67) (H, I). Aged NP effects are more pronounced but similarly reversed by AMG487. (*n* = 4). Scale bar (F): 2 mm (top) and 500 µm (bottom). Scale bar (H): 20 µm. (J) CXCL10/CXCR3 inhibition improves overall survival in ZnO NP‐exposed mice (*n* = 10). (K) Schematic diagram of pulmonary niche remodeling by ZnO NPs exposure facilitating breast cancer metastasis via CXCL10/Wnt/beta‐catenin axis. All data were represented as the mean ± SD. P values were calculated by Kaplan‐Meier analysis in J, others with one‐way ANOVA followed by Tukey's test. ^*^ *p*<0.05; ^**^ *p*<0.01.

## 3 Discussion

Studies have raised concerns regarding the potential carcinogenicity of ZnO NPs. In vitro investigations have indirectly suggested their carcinogenic potential based on the ability of ZnO NPs to induce DNA damage and genetic mutations [19, 46]. In a study utilizing *APC^min/+^* mouse model, which exhibits precancerous lesions in intestinal epithelial cells, Meng et al. demonstrated that ZnO NPs may induce malignant transformation in mouse colon tissues [47]. Our previous work demonstrated that chronic exposure of mouse embryonic fibroblasts (MEFs) to low doses of ZnO NPs led to malignant transformation, conferring upon these cells the capacity to form subcutaneous tumors in xenograft models using immunodeficient mice [20]. However, the potential influence of ZnO NPs on host‐tumor interactions and tumor growth had not yet been taken into consideration.

Given the growing body of evidence that physicochemical transformations during aging could predispose ZnO NPs to unexpected health risks, this study specifically focuses on their aged counterparts [48]. Our previous study revealed that a fraction of ZnO NPs aged in ultrapure water for ≥ 20 days transformed into Zn^2+^ ions and Zn_5_(CO_3_)_2_(OH)_6_ [19]. Furthermore, after natural aging for ≥ 60 days, the transformed ZnO NPs exhibited reduced cytotoxicity but enhanced mutagenicity in human‐hamster hybrid (A_L_) cells [20]. In line with this, Zhang et al., who investigated the role of physicochemical transformations in the aging‐dependent toxicity of ZnO NPs, also found that 30‐day‐aged ZnO NPs induced the highest toxicity in *Chlorella vulgaris* compared to either fresh nanoparticles or those aged for longer periods [8]. Similar transformations of ZnO NPs into Zn_5_(CO_3_)_5_(OH)_6_ and ZnCO_3_ were reported by Reed et al. and Sivry et al., although they did not examine the toxicity of the transformed particles [49, 50]. These studies suggest that the prevailing view regarding ZnO NPs as inherently safe materials is unsound. Furthermore, it should be noted that, to date, transformation products of ZnO NPs have been detected solely through several fundamental routine qualitative methods (TEM, XRD, dynamic light scattering, Fourier transform infrared/Raman spectroscopy). Future adoption of more precise quantitative methods (such as extended X‐ray absorption fine structure spectroscopy) will aid in evaluating the transformation process of ZnO NPs. And future work should place greater emphasis on assessing the toxicological effects of transformed NPs.

The roles of dissolution and undissolved parts in the toxicity of ZnO NPs have been studied both in vitro and in vivo [51, 52, 53]. The prevailing theory on the toxicity of ZnO NPs suggests that their effects primarily depend on the direct interaction of zinc ions with molecular targets, thereby triggering a cascade of cellular responses [54, 55, 56, 57]. Although numerous studies have emphasized that dissolved Zn^2+^ is the primary contributor to the toxicity of ZnO NPs, there is currently no consensus on this issue. Some studies have indicated that ZnO NPs suspensions, rather than the supernatant of suspensions, inhibit microbial growth, suggesting that the toxic effects are primarily caused by ZnO NPs themselves rather than dissolved Zn^2+^ [58]. Solorio‐Rodriguez et al. used high‐throughput CometChip and micronucleus (MicroFlow) assays to test the DNA and chromosomal damage caused by ZnO NPs to mouse lung epithelial cells (FE1). The results showed that the mutagenicity of ZnO NPs is jointly influenced by particle stability and chemical composition, while solubility plays an important role but is insufficient to explain the observed toxicity [59]. These conflicting observations may stem from the transformation mechanisms of ZnO NPs in the environment, the stability of the suspension, exposure methods, and the nature of the experimental models. A fundamental understanding of the scope and extent to which dissolved and undissolved particles contribute to toxicity can provide critical information for assessing potential risks to ecosystems and humans. Our previous research on the physicochemical transformations of ZnO NPs during aging and its genotoxic effects has already demonstrated that the release of zinc ions and nanoparticle uptake made significantly different contributions to the mutagenicity of fresh and aged ZnO NPs [19]. We deduced that the higher cytotoxicity of fresh ZnO NPs could be mainly due to the intrinsic toxicity of ZnO NPs, while the higher mutation potential of aged ZnO NPs might be attributed to the main contribution of Zn^2+^ and the lesser contribution from the major transformed products, i.e. Zn_5_(CO_3_)_2_(OH)_6_ [19]. Following multiple studies confirming the aging phenomenon of ZnO NPs in ultrapure water [20, 60, 61], this research shifted its focus to detecting and analyzing the capacity of aged ZnO NPs to promote cancer progression. After all, various nanomaterials have been shown to modulate tumour immunity [62, 63, 64]. Consequently, we found that, compared withWe found that, compared to fresh ZnO NPs, aged NPs exhibit a stronger ability to promote lung metastasis of breast cancer cells. Furthermore, we identified that pulmonary exposure of ZnO NPs reeducated lung TME by building an immunosuppressive and fibrotic niche, but the precise molecular targets and underlying regulatory mechanisms of ZnO NPs involved remain to be fully elucidated.

CXCL10 exhibits context‐dependent biphasic effects on the polarization of TAMs, demonstrating both pro‐ and anti‐M2 functions that are intricately shaped by TME context. Spatial transcriptomic studies in human carcinomas reveal that CXCL10 expression colocalizes with heterogeneous TAM subpopulations, where high‐CXCL10 regions contain both M2‐like and M1‐like subsets. Several studies support an anti‐M2 role for CXCL10. For instance, the BCL‐2 inhibitor APG‐2575 was shown to repolarize immunosuppressive M2 macrophages toward an immunostimulatory M1 phenotype, accompanied by elevated CXCL10 and CCL5 secretion, restored T‐cell cytotoxicity, and enhanced antitumor immunity [65]. Similarly, hedgehog signaling‐driven M2 polarization inhibits CXCL9 and CXCL10 production in TAMs, thereby impairing CD8^+^ T‐cell infiltration into the TME [66]. Conversely, compelling evidence also indicates M2‐promoting functions of CXCL10. Exosomal biglycan was reported to drive gastric cancer progression by inducing M2 polarization through CXCL10‐mediated activation of the JAK/STAT1 pathway [67]. Additionally, ALKBH5 facilitates the recruitment of PD‐L1^+^ TAMs and promotes M2 polarization via coordinated secretion of CCL2 and CXCL10 [68]. This functional duality underscores the spatiotemporal complexity of CXCL10‐mediated macrophage polarization. A comprehensive understanding of its role will require advanced single‐cell and spatial omics technologies to dynamically resolve macrophage phenotypic shifts during tumor progression and delineate the precise mechanisms through which CXCL10 modulates these processes. In our study, CXCL10 served as a key regulator in ZnO NP‐driven remodeling of TME. CXCL10, secreted by macrophages and fibroblasts, promoted breast cancer metastasis through activation of the Wnt/β‐catenin pathway. The critical finding is that pharmacological inhibition of the CXCL10/CXCR3 axis significantly attenuates the dual metastatic effects induced by both fresh and aged ZnO NPs.

In summary, our study establishes pulmonary exposure to ZnO NPs, especially aged NPs, as a potent promoter of breast cancer metastasis via re‐education of lung TME. As illustrated in Figure 6K, aged ZnO NPs induce pronounced enrichment of M2‐polarized macrophages, MDSCs, and activated CAFs, collectively fostering an immunosuppressive and pro‐fibrotic metastatic niche. We further identify CXCL10, secreted by M2 macrophages and CAFs, as a key mediator that facilitates metastatic progression through activation of the Wnt/β‐catenin signaling pathway within tumor cells. This study elucidates the cellular and molecular mechanisms of aged ZnO NP‐ induced breast cancer metastasis, underscores the necessity of mitigating exposure to ZnO NPs in ambient air, and highlights the importance of considering environmental aging processes in the risk assessment of nanomaterials.

## 4 Materials and Methods

### 4.1 Preparation of Single‐Cell RNA Sequencing Samples

To investigate alterations in the pulmonary microenvironment during breast cancer lung metastasis, mice were exposed to ZnO NPs three times followed by intravenous injection of 4T1‐luc cells. Lung tissues were collected on day 7 after injection. Single‐cell suspensions were processed using the 10x Genomics Chromium 3' v3 system (PN‐1000075). Through microfluidics, individual lung cells and barcoded gel beads were encapsulated in water‐in‐oil droplets, where cell lysis, mRNA reverse transcription, and barcoding were performed. Subsequent steps included cDNA amplification, enzymatic fragmentation, adapter ligation, and indexed PCR, resulting in the construction of single‐cell libraries for high‐throughput sequencing.

### 4.2 Single‐Cell RNA Sequencing and Analysis

Following library qualification and fragment size assessment, single‐cell RNA sequencing was performed on the 10× Genomics platform (OE Biotech Co., Ltd. Shanghai, China). Raw sequencing data (including newly generated and public datasets: GSM6687572, GSM7518067, GSM7518069) were aligned to the mouse reference genome (GRCm38) using the Cell Ranger software suite (https://support.10xgenomics.com/singlecell‐gene‐expression/software/downloads/latest). Primary processing and downstream analyses were conducted with Seurat (v3.1.0). Low‐quality cells were filtered based on the following thresholds: genes detected < 200, percentage of counts from the top gene > 90%, and mitochondrial gene ratio > 15%. Counts were normalized and highly variable genes were selected. Dimensionality reduction and visualization were performed using uniform manifold approximation and projection (UMAP). Differentially expressed genes were identified with FindConservedMarkers (logFC > 0.25, min.pct > 0.25) and used for cell type annotation based on the CellMarker database (http://biocc.hrbmu. edu.cn/CellMarker/). Pseudotime trajectory inference was carried out with Monocle2, and cell–cell communication analysis was performed using CellPhoneDB (https://github. com/ventolab/cellphonedb‐data). Gene set variation analysis (GSVA) was conducted with the GSVA package (v1.40.1). Functional enrichment analysis of GO and KEGG terms was performed on differentially expressed genes using clusterProfiler (v4.2.2).

### 4.3 Preparation and Characterization of ZnO NPs

ZnO NPs (99.9% purity, nearly spherical, diameter 20 nm) were purchased from Nanostructured & Amorphous Materials (Houston, TX) and used in the current study without any modification. ZnO nanopowder were suspended in Milli‐Q water (Millipore, 18 Mcm) to 1 mg mL^−1^ concentration and sterilized by heating to 120°C for 30 min. The suspension was stored at room temperature for naturally aging process to 60 days (referred as aged NPs). The morphology and crystallinity of both fresh and aged ZnO was characterized by transmission electron microscopy (TEM, JEOL 2100 HT, JEOL, Tokyo, Japan) and X‐ray diffraction (XRD, PANalytical B. V., Shanghai, China). The other physicochemical properties of ZnO NPs‐ including hydrodynamic size, zeta potential, PDI, and Zn^2+^ release kinetics‐ were systematically characterized in our prior studies in F‐12 medium (0, 20, 40, 60, and 120 days of aging) and DMEM medium (0 and 60 days of aging) [19, 69].

### 4.4 Cells and Cell Culture

The following cell lines were used: murine breast cancer cells (4T1), human breast cancer cells (MDA‐MB‐231, MCF‐7), murine embryonic fibroblasts (NIH‐3T3), human embryonic fibroblasts (IMR90), murine monocytic macrophages (RAW264.7), human monocytic cells (THP‐1), murine primary alveolar macrophages, murine primary MDSCs, and murine primary fibroblasts.

4T1‐luc cells, primary alveolar macrophages, and primary MDSCs were cultured in RPMI‐1640 medium (Procell, PM150110) supplemented with 10% fetal bovine serum (FBS; Procell, 164210) and 1% penicillin‐streptomycin (Biochannel, BC‐CE‐012). MDA‐MB‐231, MCF‐7, NIH‐3T3, IMR90, RAW264.7, and THP‐1 cells were maintained in DMEM (Procell, PM150210) supplemented with 10% FBS and 1% penicillin‐streptomycin.

THP‐1 cells were differentiated into macrophages by treatment with 10 ng mL^−1^ phorbol 12‐myristate 13‐acetate (PMA; Sigma, P8139) for 48 h. Murine bone marrow‐derived macrophages were differentiated into MDSCs using 40 ng mL^−1^ interleukin‐6 (IL‐6; Chamot Biotechnology, CM007‐5MP) and granulocyte–macrophage colony‐stimulating factor (GM‐CSF; Chamot Biotechnology, CM060‐5MP) for 5 days. All cells were cultured at 37 °C in a humidified atmosphere containing 5% CO_2_.

### 4.5 Animals

Female BALB/c mice (8 weeks old) were purchased from Jiangsu GemPharmatech Co., Ltd. and maintained under specific pathogen‐free (SPF) conditions in the P2‐level animal facility of the Basic Medical School, Anhui Medical University. All animal experiments were approved by the Institutional Animal Care and Ethics Committee of Anhui Medical University (LLSC 20210812).

Exposure limits of ZnO in many countries (e.g. USA, United Kingdom, Australia, Canada) were set to 5.0 mg/m^3^ (https://cdc.gov/Niosh/npg/npgd0675.html). Based on an 8 h working day, a minute ventilation volume of 6 L/min, and a standard adult body weight of 75 kg, the theoretical daily inhalation exposure is approximately 192 µg/kg/day [22]. The exposure dose in mice was selected with reference to this occupational exposure level. Considering the average body weight of the female BALB/c mice used in this study (approximately 22g), the direct pulmonary delivery route via intratracheal instillation, and the once‐weekly exposure schedule, each mouse received 30µg of ZnO NPs per administration. This dose corresponds to approximately 1.36 mg/kg per exposure. When averaged over a 7‐day period, the weekly exposure regimen corresponds to approximately 194.8 µg/kg/day, which is comparable to the estimated daily inhalation dose associated with human occupational exposure. Therefore, this exposure protocol was selected as a biologically relevant reference dose for evaluating ZnO NP‐induced pulmonary microenvironmental remodeling.

To evaluate the alterations in the pulmonary microenvironment induced by ZnO NPs, mice were randomly divided into three groups and subjected to non‐surgical intratracheal instillation of PBS, Fresh ZnO suspension, or Aged ZnO suspension (30 µg per mouse) once weekly for three consecutive exposures. Five days after the final instillation, a subset of mice was sacrificed for collection of serum and BALF. Lung tissues were harvested for hematoxylin and eosin (H&E) staining and histopathological assessment.

To determine whether pulmonary exposure to ZnO NPs promotes breast cancer lung metastasis, the remaining mice were injected intravenously with 2 × 10^5^ 4T1‐lucferase‐expressing (4T1‐luc) cells five days after the third instillation. Tumor metastasis in the lungs was monitored by in vivo bioluminescence imaging. Mice were euthanized on day 11 post‐injection, and serum, BALF, and lung tissues were collected for H&E staining and histological evaluation.

To verify whether ZnO NP‐stimulated cells in a co‐culture model facilitate breast cancer lung metastasis, RAW264.7 and NIH‐3T3 cells were pretreated with PBS, 7.5 µg/mL Fresh ZnO, or 7.5 µg mL^−1^ Aged ZnO suspensions and co‐cultured with 4T1‐luc cells for 72 h. Subsequently, 2 × 10^5^ 4T1‐luc cells were injected intravenously into mice. Tumor metastasis was assessed by bioluminescence imaging, and lung tissues were harvested, fixed, sectioned, and analyzed by H&E and immunohistochemical (IHC) staining to quantify metastatic foci.

Based on preliminary findings showing that pulmonary exposure to ZnO NPs upregulates CXCL10 expression, additional experiments were performed to test whether inhibition of CXCL10 reverses the pro‐metastatic effects of ZnO NPs exposure. Mice received intraperitoneal injections of AMG487 (MedChemExpress, HY‐15319), a CXCL10 receptor antagonist. AMG487 stock solution was prepared by dissolving the powder in DMSO, followed by sequential dilution with PEG300 (MedChemExpress, HY‐Y0873), Tween‐80 (MedChemExpress, HY‐Y1891), and finally saline. Mice were administered AMG487 at a dose of 0.4 mg per mouse one day before 4T1‐luc injection, and again on days 2, 4, and 6 post‐injection. Tumor metastasis was monitored by bioluminescence imaging, and lung tissues were collected for H&E and IHC staining to evaluate metastatic burden.

### 4.6 Serum Collection

The blood samples were collected by retro orbital bleeding, placed at room temperature for 1 h and then centrifuged (400g, 10 min, 4 °C). Serum was carefully transferred into new EP tubes, labeled, and stored at −80 °C until use.

### 4.7 In Vivo Bioluminescence Imaging

Following three intratracheal instillations of ZnO NPs, mice were intravenously injected with 2 × 10^5^ 4T1‐luc cells on day 5. Bioluminescence imaging was performed starting on day 5 post‐injection. Mice were intraperitoneally administered 100 µL of D‐luciferin solution (30 mg mL^−1^; GLPbio, GC43497), anesthetized with isoflurane, and imaged using the IVIS 100 system within 20 min.

### 4.8 Preparation of Primary Single‐Cell Suspensions from Mice

Lung tissue single‐cell suspension: Lungs from ZnO NP‐exposed mice were cut into 3–5 mm pieces and digested in 3 mL of a mixed enzymatic solution containing 2 mg mL^−1^ collagenase IV (Gibco, YL17104019) and 0.1 mg mL^−1^ DNase I (Sigma, DN25) at 37 °C in a shaking incubator for 2 h. Digestion was terminated by adding DMEM (Procell, PM150210) supplemented with 10% FBS (Procell, 164210) and 1% penicillin–streptomycin (Biochannel, BC‐CE‐012). The cell suspension was filtered through a nylon mesh, centrifuged, and resuspended in 2 mL red blood cell lysis buffer (Solarbio, R1010) on ice for 5 min. After washing with PBS and centrifugation, the resulting cell pellet was collected as the lung single‐cell suspension.

Spleen single‐cell suspension: Freshly isolated spleens were placed in pre‐chilled PBS and gently ground with the blunt end of a syringe. The homogenate was passed through a nylon mesh into a centrifuge tube. Red blood cells were lysed with 2 mL of RBC lysis buffer (Solarbio, R1010) on ice for 5 min. Cells were then washed with PBS, centrifuged, and resuspended as splenic single‐cell suspensions.

Primary bone marrow‐derived macrophages (BMDMs): Hind limbs were excised, and muscles were removed. Bone marrow cells were flushed out using a 1 mL syringe filled with RPMI‐1640 medium (Procell, PM150110) supplemented with 10% FBS and 1% penicillin–streptomycin. The flushing procedure was repeated 2–3 times. Red blood cells were lysed with 1 mL of RBC lysis buffer (Solarbio, R1010) for 2 min, followed by PBS washing and centrifugation. The resulting cells were cultured in RPMI‐1640 medium containing 10% FBS, 1% penicillin–streptomycin, 40 ng mL^−1^ IL‐6 (Chamot Biotechnology, CM007‐5MP), and 40 ng mL^−1^ GM‐CSF (Chamot Biotechnology, CM060‐5MP) for 5 days. After confirmation of successful induction into MDSCs, cells were used for downstream experiments.

Alveolar lavage fluid and macrophages: Tracheae were exposed by carefully removing surrounding tissues. A small incision was made using a fine needle, and a blunted cannula was inserted and secured with cotton thread. Approximately 1 mL of pre‐chilled PBS was slowly instilled into the trachea, incubated for 2 min, and gently aspirated back. This lavage procedure was repeated at least twice. Following centrifugation, the supernatant was collected as BALF, while the cell pellet containing alveolar macrophages was resuspended in RPMI‐1640 medium supplemented with 10% FBS and 1% penicillin‐streptomycin for further culture.

### 4.9 Flow Cytometry

Alveolar macrophages and RAW264.7 cells: Cells were processed using the fixation/permeabilization buffer kit (Elabscience, E‐CK‐A109) according to the manufacturer's protocol. After fixation at room temperature for 30–60 min in the dark, cells were permeabilized, washed, and incubated with anti‐CD206‐PE antibody (1:200; Affinity, DF4149) at 4°C for 30 min in the dark. Cells were washed with PBS, resuspended in 200 µL PBS, filtered through a 200‐mesh nylon filter, and analyzed using a Beckman CytoFLEX flow cytometer.

Lung, spleen, and primary MDSCs: Single‐cell suspensions were incubated with anti‐CD11b‐APC (1:200; BD Pharmingen, 557657), anti‐Ly6G‐Pacific Blue (1:200; BD Pharmingen, 562737), and anti‐Ly6C‐FITC (1:200; BD Pharmingen, 553104) at 4°C for 30 min in the dark. After washing and resuspension in 200 µL PBS, samples were filtered and analyzed on a Beckman CytoFLEX flow cytometer.

### 4.10 Fibroblasts

Lung single‐cell suspensions and NIH‐3T3 cells were stained with anti‐CD140a‐BV421 antibody (1:200; BioLegend, 135923) under the same conditions. After washing, resuspension, and filtration, cells were analyzed using a Beckman CytoFLEX cytometer.

### 4.11 Cell Co‐Culture

Human and murine macrophages (THP‐1, RAW264.7), fibroblasts (IMR90, NIH‐3T3), and primary murine MDSCs were resuspended in DMEM (Procell, PM150210) or RPMI‐1640 (Procell, PM150110) supplemented with 10% FBS and 1% penicillin‐streptomycin. Cells (8 × 10^4^ per insert) were seeded into the upper chambers of Transwell inserts (Corning, 3412) and treated with PBS, 7.5 µg mL^−1^ Fresh ZnO, or 7.5 µg mL^−1^ Aged ZnO suspensions. Inserts were then placed in six‐well plates containing human or murine breast cancer cells (MDA‐MB‐231, MCF‐7, or 4T1‐luc, 1 × 10^5^ cells per well). Co‐culture was performed for 72 h at 37 °C in a humidified incubator with 5% CO_2_. In parallel, primary alveolar macrophages obtained from BALF of PBS‐ or ZnO NP‐exposed mice were co‐cultured with 4T1‐luc cells under identical conditions for 72 h.

### 4.12 Migration and Invasion Assays

After co‐culture, breast cancer cells (human or murine) from the lower chambers were resuspended in serum‐free RPMI‐1640 at a density of 4 × 10^4^ cells per insert and seeded into the upper chambers of 24‐well Transwell inserts (BD, 353097), with or without Matrigel coating (BD, 356234). The lower chambers were filled with RPMI‐1640 containing 10% FBS as a chemoattractant. After 24 h, non‐migrated cells were gently washed with PBS, fixed with 4% paraformaldehyde for 10 min, and stained with crystal violet for 10 min. Cells on the upper side of the membrane were removed with cotton swabs, while migrated/invaded cells on the underside were imaged and counted in three randomly selected microscopic fields.

### 4.13 Bio‐Plex Pro Mouse Chemokine Assay and ELISA

Serum and BALF samples were collected from mice exposed to PBS or ZnO nanoparticles (three exposures), as well as from mice that received PBS or ZnO nanoparticle lung exposure followed by intravenous injection of 4T1‐luc cells for five days. Cytokine and chemokine concentrations were measured using the Bio‐Plex Pro multiplex assay on a Bio‐Plex 3D suspension array system with a 96‐well microplate format.

CXCL10 (IP‐10) levels were measured using a mouse ELISA kit (Elabscience, E‐EL‐M0021) following the manufacturer's instructions. Serum samples were thawed at room temperature and centrifuged at 1000 × g for 15 min; BALF was centrifuged at 1500 × g for 15 min on ice to remove debris; supernatants from primary alveolar macrophage cultures (maintained in serum‐free RPMI‐1640 medium for 24 h) were centrifuged at 1000 × g for 10 min. All samples underwent antibody incubation, TMB substrate development, and absorbance measurement at 450 nm. CXCL10 concentrations were calculated from a standard curve.

### 4.14 Transfection

siRNA sequences targeting CXCL10 were synthesized by Genepharma (Shanghai, China), and the specific sequences are listed in Table S1. NIH‐3T3 cells, RAW264.7 cells, or primary MDSCs were seeded into culture plates 8–16 h prior to transfection to achieve approximately 50% confluency at the time of transfection. RNA/plus complexes were prepared according to the instructions of the siRNA‐mate plus kit (Genepharma, Shanghai, China) and then added to the cells. Plates were gently agitated to ensure uniform distribution, and the cells were incubated under standard culture conditions. After 24 h, CXCL10 gene expression levels were assessed.

### 4.15 H&E Staining

Lung tissue samples were fixed in 10% neutral‐buffered formalin for 24–48 h, followed by paraffin embedding and sectioning at 4–6 µm thickness using a microtome. Sections were mounted onto glass slides. After standard deparaffinization and rehydration, staining was performed with a Hematoxylin and Eosin Staining Kit (Leagene Biotechnology, DH0008, Beijing, China) as follows: Hematoxylin Staining: Sections were incubated in Hematoxylin Solution for 3–5 min, then rinsed in running tap water. Differentiation & Blueing: Briefly immersed in 1% acid ethanol (differentiation) for 1–2 s, followed by rinsing in water (blueing) for 30 s. Eosin Counterstaining: Immersed in Eosin Solution for 1 min. Dehydration & Mounting: Sections were dehydrated through graded ethanol (70%, 95%, 100%), cleared in xylene, and mounted with neutral resin (Elabscience, E‐IR‐R109).

### 4.16 Immunofluorescence Staining

For cell immunofluorescence, 20 mm coverslips (Biosharp, BS‐18‐RC) were sterilized with 75% ethanol, flamed, and placed into 12‐well plates. Each well was coated with 1 mL of 0.01% poly‐L‐lysine (Biosharp, 25988‐63‐0) and incubated at 37°C for 30 min, followed by three washes with PBS. NIH‐3T3 cells, RAW264.7 cells, or 4T1/MCF7 cells treated with 40 ng mL^−1^ rCXCL10 were seeded onto the coverslips and cultured for 72 h. Cells were fixed with 4% paraformaldehyde for 15 min, washed with PBS, permeabilized with 0.2% Triton X‐100 (Solarbio, T8200) for 5 min, and blocked with 5% BSA (Solarbio, A8850) for 2 h. For tissue sections, slides underwent deparaffinization, rehydration, and blocking with 5% BSA for 2 h. Both coverslips and tissue sections were incubated overnight at 4°C with primary antibodies against α‐SMA (Affinity, AF1032), CD206 (Affinity, DF4149), or β‐Catenin (Proteintech, PRO‐CY‐50WY). After three washes, samples were incubated for 1 h at room temperature in the dark with Fluor488‐conjugated secondary antibody (Immunoway, RS32109), counterstained with DAPI (Beyotime, C1005) for 3 min, and mounted with antifade medium (Abbkine, BMU104). Images were acquired with a high‐resolution fluorescence microscope, and representative fields at the tumor–stroma interface were randomly selected for analysis. Antibody information are listed in Table S2.

### 4.17 Immunohistochemistry

Lung tissue sections from mice were baked at 70°C for 1 h, deparaffinized in xylene, and rehydrated through a graded ethanol series. Antigen retrieval was performed by microwaving sections in citrate‐based buffer (pH 6.0), followed by natural cooling to room temperature (RT). Endogenous peroxidase activity was blocked with 3%H_2_O_2_ (included in the UltraSensitive SP Kit, Maixin Biotech, KIT‐9720, Fuzhou, China) for 15 min. Non‐specific binding was suppressed by incubation with 5% normal goat serum (UltraSensitive SP Kit) for 40 min. Sections were then incubated overnight at 4°C with the following primary antibodies: F4/80 (Servicebio, GB11027; 1:750), Ki‐67 (Abcam, ab15580; 1:200), FSP‐1 (Bioss, bs‐1015R; 1:250), FN‐1 (BD Pharmingen, 610077; 1:250), CD206 (Affinity, DF7564; 1:250), αSMA (Affinity, AF1032; 1:500), bFGF (Proteintech, 14726‐1‐AP; 1:200), COX‐2 (Cell Signaling Technology, 12282S; 1:100). The next day, sections were brought to RT and incubated with a biotinylated goat anti‐mouse/rabbit secondary antibody (UltraSensitive^TM^ SP Kit) for 30 min. Signal was developed using a DAB Chromogen Kit (ZLI‐9018, ZSGB‐BIO, Beijing, China). Nuclei were counterstained with hematoxylin for 10 s, and sections were mounted with neutral resin (Elabscience, E‐IR‐R109). Antibody information are listed in Table S2.

### 4.18 Masson

Lung tissue sections were processed identically to the IHC protocol for baking, deparaffinization, and rehydration. Staining was performed using a Masson's Trichrome Stain Kit (Solarbio, G1340, Beijing, China) according to the manufacturer's instructions, with critical steps microscopically monitored. Nuclear Staining: Sections were covered with Weigert's Iron Hematoxylin Solution for 5 min, followed by rinsing in distilled water. Differentiation: Sections were immersed in Acid Differentiation Solution for 15 s. Blueing: Sections were treated with Masson's Blueing Solution for 5 min until complete nuclear blueing was confirmed microscopically, then rinsed thoroughly. Cytoplasm/Muscle Staining: Sections were incubated in Ponceau S Acid Fuchsin Solution for 2 min, then briefly washed in Weak Acid Working Solution (30 s). Collagen Differentiation: Excess liquid was removed, and sections were treated with Phosphomolybdic Acid Solution for 3 min until selective destaining of collagen fibers (loss of red hue) was observed microscopically. Sections were rinsed in Weak Acid Working Solution (30 s). Collagen Staining: Excess liquid was removed, and sections were stained with Aniline Blue Solution for 30 s until collagen fibers exhibited distinct blue coloration, followed by a final rinse in Weak Acid Working Solution (30 s). Dehydration & Mounting: Sections were dehydrated through an ethanol series, cleared in xylene, and mounted with neutral resin (Elabscience, E‐IR‐R109).

### 4.19 RNA‐seq and Analysis

4T1 cells were treated with 40 ng mL^−1^ recombinant CXCL10 (rCXCL10) for 72 h, and total RNA was extracted using Trizol reagent. RNA purity and concentration were assessed with a NanoDrop 2000 spectrophotometer (Thermo Scientific, USA), and RNA integrity was evaluated using an Agilent 2100 Bioanalyzer (Agilent Technologies, Santa Clara, CA, USA). RNA‐seq libraries were constructed using the VAHTS Universal V5 RNA‐seq Library Prep Kit following the manufacturer's instructions. Sequencing and bioinformatics analyses were performed by OE Biotech Co., Ltd. (Shanghai, China).

### 4.20 Quantitative Real‐Time PCR (qRT‐PCR)

Total RNA was extracted from mouse lung tissues, primary lung fibroblasts, primary alveolar macrophages, NIH‐3T3 cells, and RAW264.7 cells using Trizol reagent (GLPbio, GK20008) according to the manufacturer's protocol. RNA concentration and quality were assessed using a NanoDrop spectrophotometer. One microgram of total RNA was used as the template for reverse transcription with the 2×Q3 SYBR qPCR Master Mix kit (ToloBio, 22204‐01). The resulting cDNA was diluted 1:5 and subjected to real‐time quantitative PCR amplification using the SYBR Green method with the ToloScript All‐in‐one RT EasyMix for qPCR kit (ToloBio, 22107‐01). The mRNA levels of target genes were normalized to GAPDH expression. Primer sequences are listed in Table S1.

### 4.21 Western Blot Analysis

Proteins were extracted from cells using RIPA buffer and quantified using a protein assay kit. Equal amounts of protein were separated on 8%–15% SDS‐polyacrylamide gels and transferred to nitrocellulose membranes. β‐actin served as the internal control. Primary antibodies used included Survivin (Proteintech, PRO‐SUR‐50WY), c‐MYC (Proteintech, PRO‐CMY‐50WY), CyclinD1 (Proteintech, PRO‐CDM‐50WY), β‐Catenin (Proteintech, PRO‐CY‐50WY), GSK3β (Proteintech, PRO‐GSK‐50WY), and p‐GSK3βSer9 (Proteintech, PRO‐PGSK‐50WY), as summarized in Table S2.

### 4.22 Statistical Analyses

The in vitro and in vivo experiments in this study were all carried out for at least 3 times and the Figures showed the average results. For cell migration, invasion assays, Ki‐67/CD206 IHC calculation, RT‐qPCR, quantification analysis in immunofluorescence and flow cytometry, unpaired two‐tailed *t*‐test or one‐way anova was used to perform statistical analyzation. Mice OS rate analysis was based on Kaplan–Meier curves and log‐rank test. Pearson's analysis and Chi‐Square test were used for correlation analyses. The results with *p* values of less than 0.05 were considered to be statistically significant. Statistical significance is indicated as follows: ^*^ *p* < 0.05, ^**^ *p* < 0.01 vs control group; ^#^ *p* < 0.05, ^##^ *p* < 0.01 vs fresh NPs group.

## Author Contributions

**Yong Zhu**: writing – review and editing, writing – original draft, funding acquisition, conceptualization, formal analysis. **Siyu Wang**: methodology, investigation, software, data curation, validation, formal analysis, visualization. **Tong Lin**: methodology, investigation, visualization, software, validation. **Jingyi Zhang**: methodology, investigation. **Jingya Yang**: methodology, investigation. **Dixiang Kang**: methodology, investigation. **Wenting Zhao**: investigation, methodology. **Na Yu**: methodology, investigation. **Meimei Wang**: conceptualization, data curation, supervision, funding acquisition, project administration, writing – original draft, writing – review and editing, formal analysis.

M.W. and Y.Z. designed research; S.W., T.L., Y.Z., D.K., W.Z., and N.Y. performed research; S.W., T.L., Y.Z., and M.W. analyzed data; M.W. and Y.Z. supervised the overall study; reviewed the manuscript; M.W. and Y.Z. wrote the paper.

## Ethics Approval

The studies involving animals were reviewed and approved by the Animal Ethics Committee of Anhui Medical University (LLSC 20210812).

## Conflicts of Interest

The authors declare no conflicts of interest.

## Supporting information




**Supporting File**: advs78249‐sup‐0001‐SuppMat.docx.

## Data Availability

The data that support the findings of this study are available on request from the corresponding author. The data are not publicly available due to privacy or ethical restrictions.
