# Supplementary material for: Aged ZnO Nanoparticles Promote Breast Cancer Lung Metastasis by Remodeling the Pulmonary Microenvironment Through the CXCL10/Wnt/β‐Catenin Axis
Source: Adv Sci (Weinh). 2026 Oct 11:e78249. Online ahead of print. doi: 10.1002/advs.78249 (PMC13655249; doi:10.1002/advs.78249)
Supplement: Supplementary file 1 [file ADVS-9999-e78249-s001.docx]

**Supporting Information**

This PDF file includes:

Figures S1 to S9

Tables S1 to S3

**Figure S1.** Cell subtypes analysis of lung tissues derived from pulmonary metastasis model of breast cancer using published scRNA-seq. (A) UMAP plot showing the clustering results of 10 major cell types in whole lung tissues using published scRNA-seq datasets of breast cancer metastasis model (GSM7518067, GSM7518068, GSM7518069, GSM6687572). (B) Violin plot showing relative expression of select marker genes for each cell type shown in (A). (C) Bar plots showing proportions of cell types across different groups shown in (A). (D) Bar plot showing total cell numbers in each cell type shown in (A).

**Figure S2.** Immunosuppressive and fibrotic remodeling of the pulmonary niche in breast cancer metastasis. (A) UMAP clustering analysis of myeloid subsets using published scRNA-seq datasets of breast cancer metastasis model (GSM7518067, GSM7518068, GSM7518069, GSM6687572). (B) Violin plot showing relative expression of select marker genes for each cell type shown in (A). (C) Bar plot showing total cell numbers in each cell type shown in (A). (D) Comparison of myeloid subsets proportions in lung tissues with versus without breast cancer lung metastasis. KEGG pathway enrichment analysis of M2 macrophages (E) and MDSCs (F) in lung tissues with breast cancer lung metastasis. (G) UMAP clustering analysis of fibroblasts subsets using published scRNA-seq datasets of breast cancer metastasis model (GSM7518067, GSM7518068, GSM7518069, GSM6687572). (H) Violin plot showing relative expression of select marker genes for each cell type shown in (G). (I) Bar plot showing total cell numbers in each cell type shown in (G). (J) Comparison of fibroblasts subsets proportions in lung tissues with versus without breast cancer lung metastasis. KEGG pathway enrichment analysis of α-SMA^+^ (K) and Cd140a^+^ (L) CAFs in lung tissues with breast cancer lung metastasis.

**Figure S3.** scRNA-seq analysis of lung tissues derived from 4T1-luc tumor-bearing mice exposed to aged ZnO NPs versus control. (A) A schematic showing assessment of ZnO NPs effects on lung tissues from 4T1-luc tumor-bearing mice. (B) UMAP plot showing the clustering results of 8 major cell types in whole lung tissues derived from 4T1-luc tumor-bearing mice exposed to aged ZnO NPs versus control. (C) Violin plot showing relative expression of select marker genes for each cell type shown in (B). (D) Bar plots showing proportions of cell types across different groups shown in (B). (E) Bar plot showing total cell numbers in each cell type shown in (B).

**Figure S4.** Aged ZnO NPs remodels the pulmonary microenvironment toward a pro-inflammatory and immunosuppressive niche. (A) Representative H&E-stained lung sections of mice after triple intratracheal instillation of control, fresh or aged ZnO NPs (n=3). Scale bar: 100 μm (top) and 20 μm (bottom). (B, C) Representative immunohistochemical staining (B) and quantification (C) of F4/80- and COX2-positive cells in mouse lung sections of indicated groups (n=3). Scale bar: 100 μm (enlarged view in the bottom right-hand corner: 20 μm). (D, E) qPCR analysis of relative mRNA levels of S100A8, S100A9, TIMP1, CXCL1, CXCL9, IL-10, COX2, CD206, ARG1, Fizz1, and PPARγ in lung tissues (D) and BALF macrophages (E) (n=3). (F–I) Flow cytometry analysis of granulocytic PMN-MDSCs (CD11b^+^Ly6G^+^Ly6C^low^) and monocytic M-MDSCs (CD11b^+^Ly6C^+^Ly6G^low^) in both spleen (F, G) and lung (H, I) tissues of indicated groups (n=3). (J–M) CD206^+^ M2 macrophage polarization is confirmed by flow cytometry (J, K) and immunofluorescence (L, M) in lung sections of indicated groups (n=3). Scale bar: 100 μm. All data were represented as the mean ± SD. P values were calculated by one-way ANOVA followed by Tukey's test. **p* < 0.05, *** p* < 0.01 vs control group; *^#^ p* < 0.05, *^##^ p* < 0.01 vs fresh NPs group.

**Figure S5.** Pulmonary aged ZnO NPs exposure induces fibrotic remodeling. Mice received three weekly intratracheal instillations of Control, fresh, or aged ZnO NPs. **(**A–C**)** Masson's trichrome (A) and immunohistochemistry (B, C) reveal collagen deposition and expression of fibrotic markers (FN1, FSP-1, bFGF) in lung sections of indicated groups (n=3). Scale bar: 100 μm (enlarged view in the bottom right-hand corner: 20 μm). **(**D, E**)** qPCR analysis of relative mRNA expression levels of FN1, FSP1, bFGF, VEGFA, α-SMA, and CD140a in lung tissues (D) and primary lung fibroblasts (E) of indicated groups. (F–I) Flow cytometry and immunofluorescence demonstrate increased CD140a^+^ and α-SMA^+^ fibroblast activation in NP-exposed lungs. (F, G) Flow cytometric analysis (F) and quantification (G) of the proportion of CD140a-positive cells among primary lung fibroblasts (n=3). (H) Representative immunofluorescence staining of α-SMA in mouse lung sections (n=3). Scale bar: 100 μm. (I) Quantification of α-SMA-positive area based on immunofluorescence staining. All data were represented as the mean ± SD. P values were calculated by one-way ANOVA followed by Tukey's test. **p* < 0.05, *** p* < 0.01 vs control group; *^#^ p* < 0.05, *^##^ p* < 0.01 vs fresh NPs group.

**Figure S6.** Exposure to aged ZnO NPs promotes invasive and migratory behaviors of breast cancer cells *in vitro* co-culture models. Murine (Raw264.7 macrophages, NIH-3T3 fibroblasts) and human (THP-1 macrophages, IMR-90 fibroblasts) stromal cells were exposed to ZnO NPs (fresh or aged) and co-cultured with breast cancer cells (4T1, MCF-7, or MDA-MB-231) for 72 hours. Transwell assays show that ZnO NPs-primed macrophages (A–L) and fibroblasts (M–U) consistently enhance cancer cell migration and invasion across all species and cell line combinations, with aged ZnO NPs producing stronger effects than fresh NPs (n = 3). Scale bar: 20 μm. All data were represented as the mean ± SD. P values were calculated by one-way ANOVA followed by Tukey's test. **p* < 0.05, *** p* < 0.01 vs control group; *^#^ p* < 0.05, *^##^ p* < 0.01 vs fresh NPs group.

**Figure S7.** Aged ZnO NPs drive M2 macrophage polarization, CAF activation, and MDSC expansion *in vitro*. Murine Raw264.7 macrophages, NIH-3T3 fibroblasts, and bone marrow–derived MDSCs were exposed to ZnO NPs (fresh or aged) and co-cultured with 4T1 cells for 72 hours. (A–E) ZnO NP-primed macrophages increased CD206-positive macrophages, evidenced by flow cytometry (A, B), immunofluorescence (C, D) and mRNA upregulation of CD206 and ARG1 (E). Scale bar: 10 μm.  (F–J) ZnO NP-primed fibroblasts exhibit CAF activation, with increased CD140a^+^ and α-SMA^+^ cells and elevated fibrosis-associated genes (FN1, FSP1, α-SMA, CD140a), as evidenced by flow cytometry (F, G), immunofluorescence (H, I) and qPCR analysis (J). Scale bar: 100 μm. (K) Schematic illustration of primary MDSCs/4T1 cells co-culture system. (L, M) ZnO NP-primed MDSCs display enhanced expansion of granulocytic PMN-MDSCs (CD11b^+^Ly6G^+^Ly6C^low^). Aged ZnO NPs consistently produce stronger effects than fresh NPs across all models. Data are mean ± SD; * *p* < 0.05, ** *p* < 0.01 vs. PBS; ^#^ *p* < 0.05, ^##^ *p* < 0.01 vs. fresh NPs (one-way ANOVA with Tukey's post hoc test; n = 3 per condition).

**Figure S8.** CXCL10 activates Wnt/β-catenin signaling in breast cancer cells. (A) Principal Component Analysis (PCA) of 4T1-luc cells treated with recombinant CXCL10 (rCXCL10, 40 ng/ml) and negative control (n = 3). (B) Volcano plot of remarkable differentially expressed genes between 4T1-luc cells treated with rCXCL10 (40 ng/ml) and negative control (n = 3). (C) KEGG pathway enrichment identifying Wnt signaling as a top upregulated pathway. (D–H) qPCR and immunoblotting confirmed expression of Wnt downstream targets (c-Myc, Cyclin D1, Survivin) at both mRNA and protein levels. (I-K) Immunoblot analysis of key regulatory proteins in the Wnt signaling pathway, including β-catenin, GSK3β, and p-GSK3β (Ser9), in 4T1-luc and MCF-7 cells after rCXCL10 stimulation (n = 3). (L) Representative immunofluorescence images showing β-catenin localization in 4T1-luc and MCF-7 cells after rCXCL10 stimulation. Scale bar: 10 μm (n = 3). All data were represented as the mean ± SD. P values were calculated by one-way ANOVA followed by Tukey's test.**p* < 0.05, *** p* < 0.01 vs control group. Ns= not significant.

**Figure S9.** Pro-metastatic activity of CXCL10 is dependent on Wnt pathway activation in breast cancer cells. (A-D) Transwell assays of 4T1 (A, B) and MCF-7 (C, D) cells pretreated with ICG-001 (5 μM) for 2 h followed by stimulation with the corresponding rCXCL10 (50 ng/ml ) for 24 h ( n = 3 ). Scale bar: 20 μm. All data were represented as the mean ± SD. P values were calculated one-way ANOVA followed by Tukey's test. **p* < 0.05, *** p* < 0.01 vs control + DMSO group; *^#^ p* < 0.05, *^##^ p* < 0.01 vs fresh rCXCL10 + DMSO group.

**Table S1. Sequences of siRNAs and primers for qRT-PCR**

| **Gene Name** | **Forward Primer Sequence (5'→3')** | **Reverse Primer Sequence (5'→3')** |
| --- | --- | --- |
| GADPH | AAATGGTGAAGGTCGGTGTG | AGGTCAATGAAGGGGTCGTT |
| S100A8 | TCGTGACAATGCCGTCTGAA | GACATATCCAGGGACCCAGC |
| S100A9 | AGATGGCCAACAAAGCACCT | TAGACTTGGTTGGGCAGCTG |
| TIMP1 | CGAGACCACCTTATACCAGCG | ATGACTGGGGTGTAGGCGTA |
| CXCL1 | CAATGAGCTGCGCTGTCAGT | TTGAGGTGAATCCCAGCCAT |
| CXCL9 | GAACCCTAGTGATAAGGAATGC | TGAGGTCTTTGAGGGATTTGTAG |
| IL10 | CAAGGAGCATTTGAATTCCC | GGCCTTGTAGACACCTTGGTC |
| COX2 | TATCAGGTCATTGGTGGAG | AGGGATGTGAGGAGGGT |
| CD206 | CTCTGTTCAGCTATTGGACGC | CGGAATTTCTGGGATTCAGCTTC |
| ARG1 | AAGAATGGAAGAGTCAGTGTGG | GGGAGTGTTGATGTCAGTGTG |
| Fizz1 | GAACTTCTTGCCAATCCA | GTCCAGTCAACGAGTAAG |
| PPARγ | GTCACGTTCTGACAGGACTGTGTGAC | TATCACTGGAGATCTCCGCCAACAGC |
| FN1 | CAACAGACCACCAAACTCG | CACAACAATGCTCCCAGA |
| FSP1 | GATGTAATTGTGTCCACCTTC | GCTGTCCAAGTTGCTCAT |
| bFGF | CGAGAAGAGCGACCCA | TTTCAGTGCCACATACCAA |
| VEGFA | CTACTGCCGTCCGATTGA | GTGAACGCTCCAGGATTTA |
| α-SMA | ATCACCATCGGAAATGAACG | CTGGAAGGTGGACAGAGAGG |
| CD140a | GGAGAACCTGTTGCCGGGAC | TCTCGATGGCACTCTCTTCC |
| Survivin (mouse) | GAACCCGATGACAACCCGAT | TGGTCTCCTTTGCAATTTTGTTCT |
| c-MYC (mouse) | TGCGGTGACTGATATACGCA | ACCATTTTCTCTTGCTCGCG |
| CyclinD1 (mouse) | GAGAAGTTGTGCATCTACACTG | AAATGAACTTCACATCTGTGGC |
| Survivin (human) | CATTCGTCCGGTTGCGCTTTCC | GCGCACTTTCTCCGCAGTTTCC |
| c-MYC (human) | GGCTCCTGGCAAAAGGTCA | CTGCGTAGTTGTGCTGATGT |
| CyclinD1 (human) | GAGAAGTTGTGCATCTACACTG | AAATGAACTTCACATCTGTGGC |
| CXCL10 | CCAAGTGCTGCCGTCATTTTC | GGCTCGCAGGGATGATTTCAA |
| Si-CXCL10-1 | GCAACUGCAUCCAUAUCGATT | UCGAUAUGGAUGCAGUUGCTT |
| Si-CXCL10-2 | GAAGCUUGAAAUCAUCCCUTT | AGGGAUGAUUUCAAGCUUCTT |
| Si-CXCL10-3 | CGUGUUGAGAUCAUUGCCATT | UGGCAAUGAUCUCAACACGTT |

**Table S2. Antibody Information**

| **Antibody** | | **Application** | | **Host Species** | **Dilution Ratio** | **Catalog Number** |
| --- | --- | --- | --- | --- | --- | --- |
|  | Ly-6C-FITC | | FACS | Mouse | 1:200 | BD Pharmingen, 553104 |
|  | LY-6G-BV421 | | FACS | Mouse | 1:200 | BD Pharmingen, 562737 |
|  | CD11b-APC-Cy7 | | FACS | RAT | 1:200 | BD Pharmingen, 557657 |
|  | CD140a-BV421 | | FACS | Mouse | 1:200 | biolegend, 135923 |
|  | MRC1/CD206 | | IF/IHC | Mouse | 1:200 | Affinity, DF4149 |
|  | a-SMA antibody | | IF/IKC | Mouse | 1:200 | Affinity, AF1032 |
|  | β-Catenin | | WB/IF | Rabbit | 1:10000 / 1:200 | Proteintech, PRO-CY-50WY |
|  | GSK3β | | WB | Rabbit | 1:10000 | Proteintech, PRO-GSK-50WY |
|  | p-GSK3β^Ser9^ | | WB | Rabbit | 1:10000 | Proteintech, PRO-PGSK-50WY |
|  | c-MYC | | WB | Rabbit | 1:10000 | Proteintech, PRO-CMY-50WY |
|  | Cyclin D1 | | WB | Mouse | 1:10000 | Proteintech, PRO-CDM-50WY |
|  | Survivin | | WB | Rabbit | 1:1000 | Proteintech, PRO-SUR-50WY |
|  | CD206-PE | | FACS | Mouse | 1:200 | BD 568273 |
|  | F4/80 | | IHC | Mouse | 1:750 | Servicebio, GB11027 |
|  | Ki-67 | | IHC | Mouse | 1:200 | Abcam, ab15580 |
|  | Fsp1 | | IHC | Mouse | 1:250 | Bioss, bs-1015R |
|  | FN1 | | IHC | Mouse | 1:250 | BD Pharmingen, 610077 |
|  | bFGF | | IHC | Mouse | 1:200 | Proteintech, 14726-1-AP |
|  | COX2 | | IHC | Mouse | 1:100 | Cell Signaling Technology, 12282S |
|  | Alexa Fluor® 488-conjugated goat anti-rabbit IgG | | IF/ICC | rabbit | 1:200 | RS32109 |
|  | DDDDK-Tag (binds to flag sequnence) (2C5) Mouse mAb (AbFluor 488) | | IF | Mouse | 1:200 | Immunoway, YM2030 |

**Table S3.** **Reagents and Consumables**

| **Reagents / Consumables** | **Manufacturer** | **Catalog Number** |
| --- | --- | --- |
| Trizol Reagent | GLPbio | GK20008 |
| RPMI 1640 Medium | Procell | PM150110 |
| High-Glucose DMEM | Procell | PM150210 |
| D-Luciferin | GLPbio | GC43497 |
| Mouse CXCL10 (IP-10) ELISA Kit | Elabscience | E-EL-M0021 |
| 24-Well Transwell Inserts | BD | 353097 |
| 24 mm Transwell Inserts with 0.4 μm Polycarbonate Membrane | Corning | 3412 |
| Fetal Bovine Serum (FBS, Premium Grade) | Procell | 164210-500ml |
| ToloScript All-in-One RT EasyMix for qPCR | ToloBio | 22107 |
| 2× Q3 SYBR qPCR Master Mix (Universal) | ToloBio | 22204-01 |
| Penicillin-Streptomycin-Amphotericin B Solution (100 ×) | Biochannel | BC-CE-012 |
| DNase I | Sigma | DN25 |
| Collagenase IV | Gibco | YL17104019 |
| DAPI | Beyotime | C1005 |
| Crystal violet | Solarbio | C8470 |
| 100× Triton X-100 | Solarbio | T8200 |
| Mouse CXCL10 (IP-10) recombinant protein | Proptech | 250-16-25UG |
| Red Blood Cell Lysis Buffer | Solarbio | R1010 |
| Poly-L-Lysine | Biosharp | 25988-63-0 |
| AMG487 | MCE | HY-15319 |
| Cell climbing coverslips | Biosharp | BS-18-RC |
| GM-CSF | Chamot Biotechnology | CM060-5MP |
| IL-6 | Chamot Biotechnology | CM007-5MP |
| Intracellular Fixation/Permeabilization Kit | Elabscience | E-CK-A109 |
| Matrigel | BD | 356234 |
| **Hematoxylin and Eosin Staining Kit** | **Leagene Biotechnology** | **DH0008** |
| **Neutral resin** | **Elabscience** | **E-IR-R109** |
| **UltraSensitive™ SP Kit** | **Maixin Biotech** | **KIT-9720** |
| **DAB Chromogen Kit** | **ZSGB-BIO** | **ZLI-9018** |
| **Masson’s Trichrome Stain Kit** | **Solarbio** | **G1340** |
